# Supplementary material for: Inverse design of stochastic, voxelated thermo-viscoelastic digital materials
Source: Nat Commun. 2025 Nov 28;16:11705. doi: 10.1038/s41467-025-66744-2 (PMC12753674; doi:10.1038/s41467-025-66744-2)
Supplement: Supplementary file 1 — Supplementary Information [file 41467_2025_66744_MOESM1_ESM.pdf]

Supplementary Information – Inverse Design of Stochastic, Voxelated Thermo-Viscoelastic Digital Materials

Marc Wirth[a,*,†], *Joël N. Chapuis*[a,†] *and Kristina Shea*[a]

[a] Engineering Design and Computing Laboratory, Department of Mechanical and Process Engineering, ETH Zurich, Tannenstrasse 3, 8092 Zurich, Switzerland.

* Corresponding author. *E-mail address:* wirthma@ethz.ch (M. Wirth).

† These authors contributed equally to this work.

Table of Contents

A. Supplementary Methods *3*

A.1 Experimental Material Characterization **3**

A.1.1 Experimental Procedure 3

A.1.2 Material Model and Data Processing 5

A.2 Voxel Structure Generation Framework **7**

A.2.1 Overview 7

A.2.2 User Inputs 8

A.2.3 Loading Input Geometry 9

A.2.4 A – Coarse Grid Generation 9

A.2.5 B – Digital Material Assignment Concept 10

A.2.6 B – Digital Material Assignment Operations 10

A.2.7 C – Master Curve Estimation 11

A.2.8 D – Prony Series Fitting Procedure 12

A.2.9 E – FE Mesh Generation 12

A.2.10 F – PNG Creation 12

A.3 Neural Network Training **14**

A.4 Orthosis Details **18**

A.4.1 Orthosis Requirements 18

A.4.2 Orthosis Design 18

A.4.3 Orthosis Simulation 20

A.4.4 Orthosis Deformation Test 23

A.5 Damper Details **24**

A.5.1 Damper Design 24

A.5.2 Hysteresis Simulation 25

A.5.3 Hysteresis Test 27

B. Supplementary Discussion *28*

B.1 Runtime Evaluation **28**

B.2 Assessment of Percolation Threshold **28**

B.3 Design and Manufacturing Repeatability **29**

B.4 Neural Network Prediction Accuracy **31**

B.5 Orthosis Fatigue Testing **32**

C. Supplementary Figures 33
C.1 Prony Series Fitting 33
C.2 Damper Simulation Results 35
C.3 Orthosis Simulation Results 36
D. Supplementary Tables 41
D.1 Fitted Material Models 41
E. Supplementary References 44

A. Supplementary Methods

A.1 Experimental Material Characterization

All samples are additively manufactured (AM) on a Stratasys J850 Prime (Stratasys Ltd., Eden Prairie, MN, USA) utilizing the inbuilt voxel AM capabilities. The manufacturing is performed with a layer height of 0.027mm and a resolution of 600 and 300dpi in *x* and *y* direction respectively. The input for the AM geometries is provided as a set of PNG files, where each pixel color corresponds to a base material of the user's choice. To account for the imbalance in planar resolution, the PNG pixels are repeated once in *x* direction. This results in an effective planar resolution of 300 dpi. All samples are additively manufactured using the base materials Agilus30Black (AB) and VeroUltraWhite (VW). Support is created using SUP706. The parts are additively manufactured at the top left corner of the print bed to ensure similar UV curing conditions[1]. The support is removed mechanically and using pressured water. The parts are stored for at least 24h before further testing.

A.1.1 Experimental Procedure

The mechanical characterization of voxelated stochastic digital material is performed using a dynamical mechanical analysis (DMA) at varying temperatures and frequencies.

A.1.1.1 Sample Design

The samples are type IV dobgones according to ASTM D638. The dogbone center is assigned the digital material with the stochastic base material mixture under test. The dogbone ends, which are clamped in the testing machine grips, are assigned VW to prevent sample slipping. A linear material gradient of 2mm length is added as a transition between the VW ends and the digital material to prevent stress concentrations (see Supplementary Figure 1).

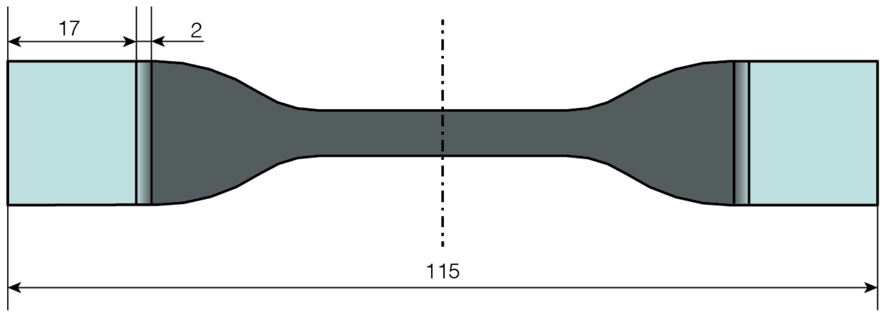

Supplementary Figure 1 Geometry of the ASTM D638 type IV dogbone samples. The core is assigned the digital material under test. The ends are assigned VeroUltraWhite. A 2mm material gradient connects the core to the VW ends.

A.1.1.2 Test Setup

All samples are tested on an Instron ElectroPuls E3000 (Instron, Norwood, MA, USA) using an Instron Dynacell 2527 with a range of ±5kN. The temperature is controlled by an Instron 3119-600 series temperature-controlled chamber using liquid nitrogen (LN_2) as a cooling agent. The LN_2 inlet pressure is set to 1.3bars.

A.1.1.3 Test Procedure

The experimental procedure used is dynamical mechanical testing, where the reaction force for a sinusoidal displacement load is measured. The measurement is performed by applying a sinusoidal displacement for each frequency and temperature, waiting for the reaction force to stabilize between individual load cycles and measuring the storage modulus E', the loss modulus E'' and the material damping $tan(\delta)$, where δ is the quotient between E' and E''. The testing follows a nested loop approach. A schematic overview of the testing procedure is seen in Supplementary Figure 2. The testing is performed in three stages indicated by i controlled by the outermost loop. The three stages control the pre-strain ε_i and the temperature step size T_i. Stage 0 corresponds to the samples being in their glassy phase. The

temperature is below the estimated glass transition temperature and the temperature step size is large since changes over temperature are expected to be less prominent. Stage 1 corresponds to the sample transition between the glassy and rubbery state. The temperature is around the estimated glass transition temperature and the temperature step is small since large changes in stiffness are expected. Stage 2 corresponds to samples in the rubbery state. The temperature is above the estimated glass transition temperature and the temperature steps are large again since no significant change in stiffness is expected. The middle loop increases the temperature by the currently set temperature step T_i. The innermost loop goes through all testing frequencies from $1Hz$ to $f_{max} = 18Hz$ in $1Hz$ steps and performs measurements for each frequency. The data is saved when the maximum temperature T_{max} is reached.

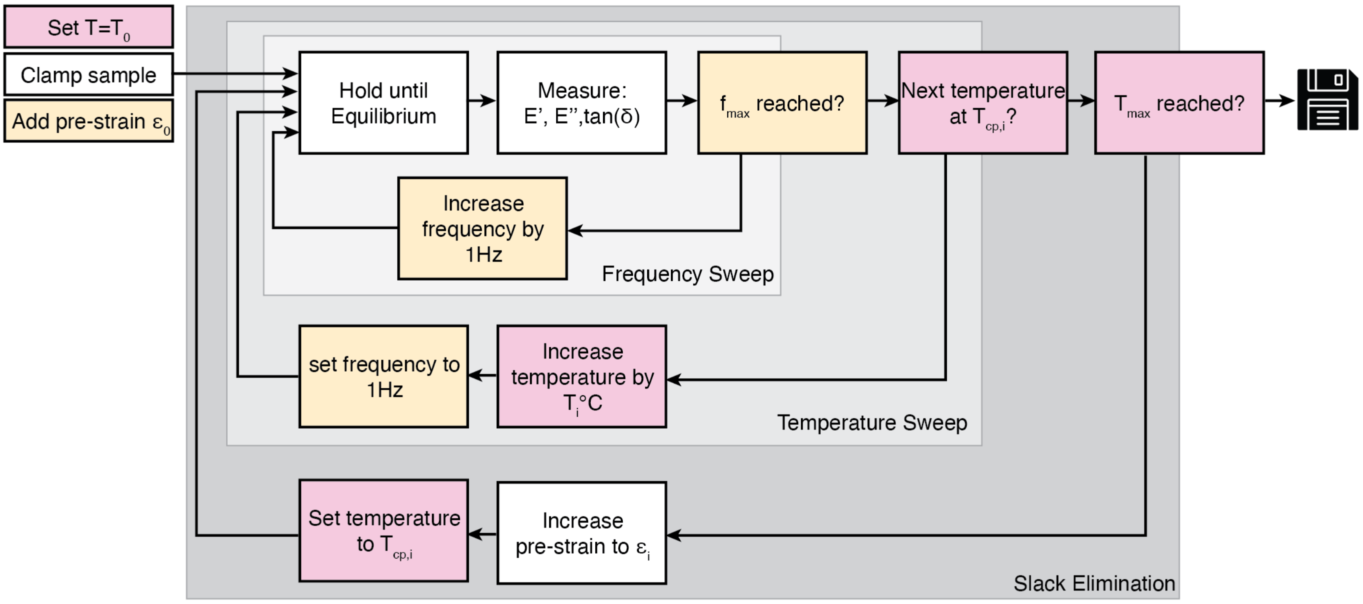

Supplementary Figure 2 Mechanical testing procedure of the dogbone samples. The test procedure is a nested loop, which measures E', E'' and $tan(\delta)$ for each frequency in the set range and for each temperature in the set range. The temperature steps and the pre-strain applied to the sample to eliminate slack are adjusted in the outer loop based on the current test stage.

A.1.1.4 Test Parameters

Due to the time cost associated with DMA testing, only one to two samples of each digital material are tested. For the base materials Agilus30Black and VeroUltraWhite, two and three samples are tested respectively. All samples are tested in all three stages except the pure Agilus30Black sample. This sample is tested only in the transition and rubbery stage as it does not reach its glassy phase in the tested temperature range. All test parameters can be found in Supplementary Table 1.

Supplementary Table 1 Test parameters of the DMA testing. All samples are tested in all three stages (glassy, transition, rubbery) except the pure Agilus30Black sample.

				**Stage i=0**			**Stage i=1**			**Stage i=2**		
%AB	%VW	n_{sample}	T_{range} [°C]	T_{cp} [°C]	ε_0 [-]	T_0 [°C]	T_{cp} [°C]	ε_0 [-]	T_0 [°C]	T_{cp} [°C]	ε_0 [-]	T_0 [°C]
100	0	2	[-13,30]	-	-	-	-	0.035	5	25	0.035	5
83.5	16.5	2	[-15,30]		0.01	5	0	0.02	5	10	0.035	5
66.6	33.3	1	[-13,80]	-	0.01	5	40	0.015	5	55	0.035	5
50	50	1	[-21,90]	-	0.01	10	45	0.015	5	70	0.035	10
33.3	66.6	1	[-13,80]	-	0.01	5	55	0.015	5	65	0.035	5
16.5	83.5	1	[-13,90]		0.01	10	50	0.015	5	80	0.035	5
0	100	3	[-13,100]	-	0.01	5	55	0.015	5	90	0.035	5

A.1.2 Material Model and Data Processing

All samples are tested between 1Hz and 18 Hz as described in Section A.1.1.3. An initial evaluation of the experimental data shows that the Instron ElectroPuls E3000 exhibits an eigenfrequency around 17Hz. This makes the measured data around this frequency unusable as the results are falsified by the resonance of the machine. The sample data is preprocessed to include the results only between 1Hz and 15Hz. The glass transition temperature T_g is derived from the observed initial peak in $tan(\delta)$ (first column Supplementary Figure 27). The continuous relationship between T_g and the base material composition is fit with a de Gennes percolation curve (see main text). The critical percolation threshold ρ_c is 0.163 and the percolation coefficient μ is 0.5547. The shift factors for the time-temperature superposition principle (TTSP) are split into two groups. Above and at T_g, the WLF equation is used and its parameters C_1 and C_2 are fixed to 17.44 and 51.6 respectively according to Ferry[2]. Below T_g, the Arrhenius function is used (see Supplementary Equation (1), Supplementary Figure 3b) and its parameter C_3 is determined based on an optimization approach applied to the TTSP.

$$\log_{10} \alpha_T = \begin{cases} C_3\left(\frac{1}{T}-\frac{1}{T_0}\right) & if\ T < T_g \quad \text{Arrhenius} \\ \frac{-C_1(T-T_0)}{C_2+(T-T_0)} & if\ T \geq T_g \quad \text{WLF} \end{cases} \tag{1}$$

The shifting procedure used to determine C_3 of the two base materials is conducted using a numerical optimization scheme based on a Nonuniform Pattern Search (NUPS) algorithm in MATLABs inbuilt function `patternsearch`. The objective is to shift the individual frequency sweeps to create a master curve that is as smooth as possible by minimizing the absolute second derivative along its entire length (second column Supplementary Figure 27). This is achieved by fitting a spline through the individual data points gathered during the mechanical testing procedure and numerically determining the second derivative as shown in Supplementary Equation (2).

$$\min_{C_3} \sum_i \int_{f_{min}}^{f_{max}} \left| \frac{d^2 E'_{exp}(C_3, f)}{df^2} \right| \tag{2}$$
$$s.t. \quad C_{\text{LB}} \leq C_3 \leq C_{\text{UB}}$$

The constant C_3 of the five digital materials is interpolated with the percolation curve fitted for the glass transition temperature T_g and the C_3 of the two base materials. A Generalized Maxwell Model consisting of $n = 31$ Maxwell elements is fitted to the experimental data. The fitting procedure is conducted in the frequency domain using a numerical optimization scheme based on a nonlinear least-squares algorithm (Supplementary Equation (3)). To improve performance and convergence during optimization, the optimizer sets the coefficient τ_n and the exponent τexp_n of the individual relaxation times separately.

$$\min_{k_n, \tau_n, \tau exp_n} \sum_{\log f} \left(E'_{exp}(\log f) - \left(k_0 + \sum_n \frac{k_n \cdot \left(10^{\log f}\right)^2 \cdot (\tau_n \cdot 10^{\tau exp_n})^2}{1 + (10^{\log f})^2 \cdot (\tau_n \cdot 10^{\tau exp_n})^2} \right) \right)^2$$
$$s.t. \quad 1 \leq k_n \leq \text{Inf}$$
$$1 \leq \tau_n \leq 10 \tag{3}$$
$$\min(\log f) - 5 \leq \tau exp_n \leq \max(\log f) + 5$$

The fitted models are expressed using a normalized Prony series. The stiffness of the Maxwell elements is thus defined as a fraction of the long-term stiffness k_0 (third column Supplementary Figure 26, Supplementary Figure 27 and Supplementary Table 7). In the final simulation model, the Hookean element k_0 that models long-term stiffness is replaced with a Mooney-Rivlin hyperelastic element (Supplementary Table 6). In combination with the normalized Prony series, this enables the simulation of larger strain ranges.

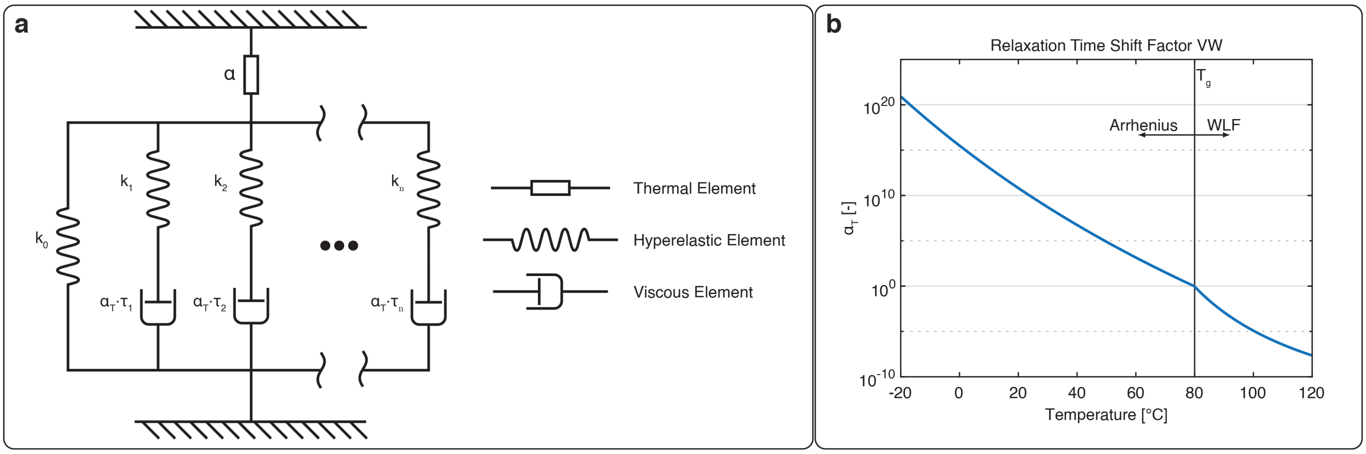

Supplementary Figure 3 (a) Illustration of the generalized Maxwell model used to represent the material behavior of the stochastic polymer mixes. The model consists of a Mooney-Rivlin hyperelastic element k_0 and n Maxwell elements consisting of a Mooney-Rivlin hyperelastic element k_n and a Newtonian dashpot with a relaxation time of τ_n. (b) Illustrative example of the combined WLF and Arrhenius model used to modulate relaxation times.

A.2 Voxel Structure Generation Framework

A.2.1 Overview

The macroscale structures with voxelated stochastic digital material are generated using a custom framework[3] programmed in MATLAB R2024a (The MathWorks, Inc., Natick, MA, USA). The framework processes the macroscale structure given as STL geometry into a voxelated structure that can be exported as a stack of PNGs for manufacturing by polymer material jetting. It also transforms the structure into a simulation model to be used in ABAQUS CAE (Dassault Systemes, Vélizy-Villacoublay, France). In Supplementary Figure 4, an overview of the framework is presented. The individual steps will be detailed in the following chapters. The framework is initialized by providing an STL and user inputs. The STL file is transformed into a 3D grid structure approximating the macroscale geometry with a mesoscale coarse grid according to a user defined resolution (Supplementary Figure 4_A). A series of basic operations is applied sequentially to assign digital materials defined as a material mixture of additively manufacturable base materials (Supplementary Figure 4_B). The coarse grid with assigned digital materials is the starting point for three processing branches. On a material property level, the master curve for each individual digital material, effectively providing the material stiffness over time, is estimated using a trained artificial neural network (ANN) (Supplementary Figure 4_C). A Prony series is fitted to the estimated master curve (Supplementary Figure 4_D). The set of Prony series for all digital materials is provided as an output which is usable as a material input in ABAQUS CAE. The coarse grid with assigned digital materials is also transformed into a meshed finite element (FE) structure with a user-defined element size (Supplementary Figure 4_E). Here, the original geometry provided by the STL file is approximated by either tetrahedral volume or triangular shell elements. The FE elements and the material data from Supplementary Figure 4_D are written to an ABAQUS input file. Further, the framework generates a voxelated microscale representation according to the printer resolution based on the structure geometry and the digital material assignment. Here, the framework interpolates the digital material for each microscale voxel based on the assigned digital material on the mesoscale coarse grid. It then assigns base material stochastically to each microscale voxel (Supplementary Figure 4_F). The microscale grid is sliced and saved as a collection of PNG files which can be additively manufactured using polymer material jetting.

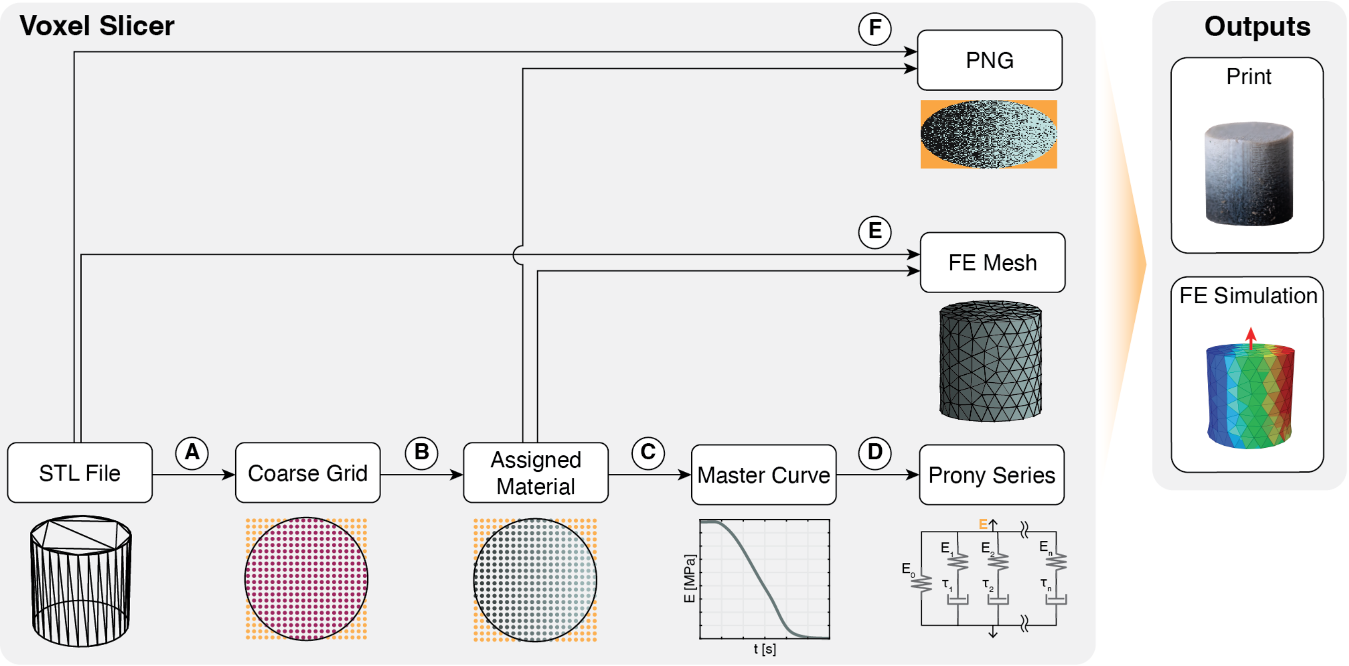

Supplementary Figure 4 Schematic representation of the framework to generate structures of voxelated digital material. (A) The STL file is loaded and transformed into a mesoscale coarse grid structure. (B) Digital material assignment operations are applied. (C) For each digital material in the coarse grid, the master curve at room temperature is evaluated using an ANN. (D) For each digital material in the coarse grid, the Prony series elements are fitted to the estimated master curve. (E) The STL file is transformed into a uniform tetrahedral solid or triangular shell mesh and assigned digital materials based on the coarse grid representation. (F) The STL file is sliced and assigned base material voxels based on the digital material defined in the coarse grid.

A.2.2 User Inputs

The framework requires user input describing the structure geometry, the manufacturing requirements, the output FE mesh, the available base materials, the coarse grid resolution, and the digital material assignment operations. A list of all inputs is provided in Supplementary Table 2. The geometry is described by an STL file and geometry type. The geometry type can be switched depending on whether the geometry is a solid or surface model. Depending on the type, the coarse grid generation and the FE mesh generation are performed differently. The geometry can be rotated after loading to account for the print direction. The manufacturing requirements are provided using the printer's resolution in build direction (h_{layer}) and in the print plane (dpi). The mesh settings are provided as FE element size L_{el}, FE element thickness t and FE field temperature T_{field}. The element thickness t is only required for *shell* type geometries. The voxel slicer is set up to create a uniform mesh of tetrahedral and triangular elements for *solid* and *shell* type geometries respectively. If wished, a custom mesh with the same element types can be provided using a MATLAB struct containing nodes and elements. The digital material information is provided as a set of base materials. For each base material, the framework requires a material ID (integer number), a material name (string) and the display color (RGB triplet). An initial digital material for the loaded geometry needs to be provided using one of three methods used to define digital materials (see Section A.2.5). The digital material assignment takes place on a mesoscale coarse grid, which is defined by the coarse grid resolution CG_{res}, defined in the three global coordinate directions. Finally, the framework requires an array of operations which are applied on the geometry to assign digital material.

Supplementary Table 2 Description of user inputs required to generate voxelated structures.

Group	Name	Type	Unit	Description
Geometry	STL	.stl	-	STL file of geometry.
Geometry	$type$	string	-	Determines if geometry is a *solid* or *shell* structure.
Geometry	R	[1 x 3]	deg	Rotation of geometry performed after import provided as an angle about the global coordinate axis in degrees.
Manufacturing	h_{layer}	[1 x 1]	mm	Layer height as specified by the printer.
Manufacturing	dpi	[1 x 1]	1/inch	*x*-*y*-resolution of the printer.
Mesh	t	[1 x 1]	mm	Thickness of FE elements. Only applicable for *shell* structures.
Mesh	L_{el}	[1 x 1]	mm	Target size for FE elements.
Mesh	$FEmesh$	string	-	Name of custom FE mesh saved as MATLAB struct containing nodes and elements.
Mesh	T_{field}	[1 x 1]	°C	Initial field temperature of FE simulation.
Material	M_{ID}	[1 x 1]	-	Integer providing a unique ID for each provided base material.
Material	M_{Name}	string	-	Name of each provided base material.
Material	M_{Color}	[1 x 1]	-	RGB triplet for each provided base material.
Material	M_{Init}	[1 x 1]<br>[n x 2]<br>[n x 3]	ID<br>[ID, ρ_{ID}]<br>[°C, s, MPa]	Initial digital material assigned to the whole geometry. Provided either by base material ID, by a mixture of any base materials or as set of requirement triplets.
Grid Information	CG_{res}	[1 x 3]	mm	Resolution of coarse grid in *x*-, *y*- and *z*-direction.
Operation	OP	{n x 1}	-	Cell array of operations, where each operation is a struct containing operation-specific instructions.

A.2.3 Loading Input Geometry

The input geometry is provided as an STL file. It is used for three purposes: determining the coarse grid by providing the size of the geometry, creating the PNG images and creating the FE mesh. For watertight STL files (type *solid*), the STL file input is not further processed. It is read by an STL reader and passed as points and edges to further functions. For open-surface STL files (type *shell*), the file is augmented to transform the 2D surface into a 3D hull (Supplementary Figure 5). The STL file is loaded, and the open edges are found. The holes are closed by new facets connected to the centroid of the edge. The closed geometry is meshed with tetrahedral elements using MATLABs inbuilt meshing algorithm `generateMesh`. Only the tetrahedral facets close to the STL surface are kept, which results in a uniform triangular mesh. The newly created uniform triangular mesh is thickened along its normal direction according to the element thickness t.

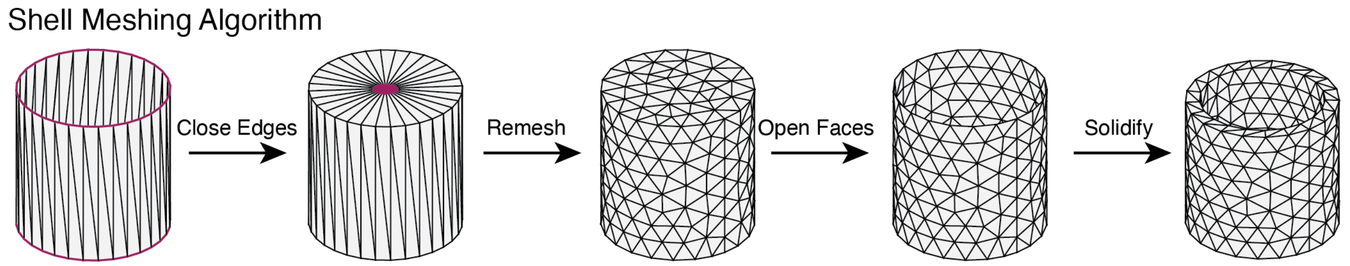

Supplementary Figure 5 Visualization of algorithm used to process open-surface STL files. The geometry is made watertight by closing the open edges, re-meshed and opened again. It is solidified along the normal direction to create a 3D approximation of the surface representation.

A.2.4 A – Coarse Grid Generation

The mesoscale coarse grid is generated in two steps: grid generation and grid filling. During grid generation, a uniformly spaced grid with variable spacing in each coordinate direction according to CG_{res} is created. The grid is created with the minimum number of points necessary in each direction, such that the bounding box of the loaded STL file is fully enclosed. During grid filling, it is determined which grid points are within the STL geometry. For *solid* type geometries, this is performed using a modified version of the voxelization algorithm by A. Aitkenhead[4], which is based on the work of Patil *et al.*[5]. For *shell* type geometries, the assignment is performed such that all coarse grid points in the vicinity of the centroids of the triangular shell mesh are indicated as being within the structure. Finally, the coarse grid points determined not to be within the structure are assigned to a void material. The coarse grid points within the structure are assigned the initial digital material provided by the user.

In practice, the coarse grid resolution is adapted to the size of the geometric features in the structure, the desired material assignment resolution and the FE mesh resolution required for simulation.

- **Geometric Feature Size:** If the part geometry contains small features, the coarse grid should be chosen such that the smallest feature can be represented by at least one coarse grid point. In principle, the resolution for homogenous material assignment can be chosen to be lower, however this severely limits the potential for the creation of graded transitions and local stiffness tuning.
- **Material Assignment Resolution:** As the materials are assigned based on the coarse grid and interpolated between the coarse grid points, the coarse grid mesh resolution should be chosen such that it matches the desired material assignment fidelity.
- **FE Mesh Resolution:** FE element materials are assigned based on the closest coarse grid point relative to the element centroid. The resolution of the FE mesh and coarse grid need to be matched, especially in regions of significant local material transitions. This ensures that the FE model can accurately represent local material gradients, and it also ensures that these gradients have adequate resolution in the coarse grid to enable an accurate export for fabrication.

A.2.5 B – Digital Material Assignment Concept

Digital material is assigned on the coarse grid as percentages of base material at each coarse grid point (see Supplementary Figure 6). Each point of the coarse grid is assigned a row in a $[n \times m]$ digital material matrix, where n is the number of grid points and m is the number of base materials plus 1. The first column is designated for the void material, which by default can only take the values of 0 and 100%.

During digital material assignment, the target digital material can be input in three different ways; *direct assignment*, *determined mixture and inverse assignment*. In *direct assignment*, the digital material is assigned a homogenous base material directly based on the provided base material ID. In *determined mixture*, the target digital material is provided as an $[i \times 2]$ matrix, where i is the number of base materials in the mixture. The two columns correspond to the base material IDs and the mixture ratios respectively. The sum of all ratios must be one. In *inverse assignment*, the digital material is provided as a set of requirement triplets in a $[j \times 3]$ matrix, where j is the number of triplets, and the columns correspond to the target temperatures, characteristic times and stiffness values respectively. In this case, the requirements tuples are extended by interpolated requirement values, such that there are eight requirement tuples linearly interpolated between each pair of input tuples. This yields a total of $8 \cdot j$ virtual requirement tuples. The virtual tuples are given to an ANN that estimates digital material constituted from Agilus30Black (AB) and VeroUltraWhite (VW) for each virtual requirement tuple. The mean estimated digital mixture is then translated to a row vector to be integrated into the digital material matrix.

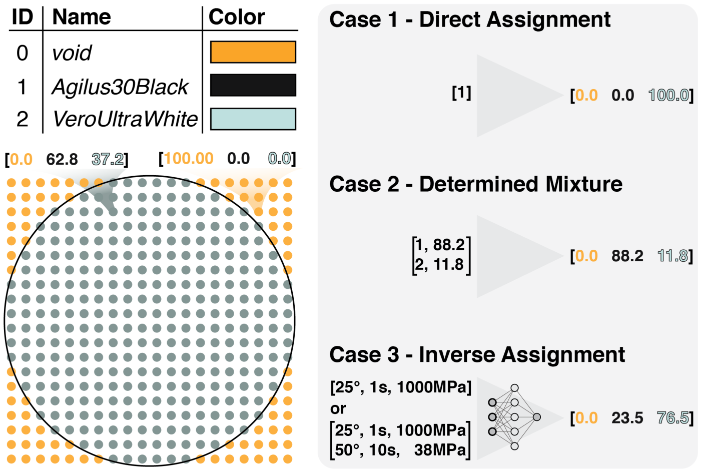

Supplementary Figure 6 Digital material assignment procedure on the coarse grid. User input defines the set of base materials, their IDs and colors. Each point in the coarse grid is assigned a digital material mixture of the base materials. The assignment can be performed in one of three cases, either by assigning one base material, by assigning a defined base material mixture or by assigning an estimate of an AB/VW mixture based on a set of input requirements.

A.2.6 B – Digital Material Assignment Operations

The digital material assignment is based on applying geometric operations assigning digital material to a set of coarse grid points. Chaining a series of operations enables the creation of a large variety of designs. Five operations are integrated in the current framework. However, there is the potential to add more operations if needed in the future.

A.2.6.1 Operation – Split

The split operation splits the whole provided geometry into two sides with two provided digital materials. The operation requires a split direction corresponding to one of the axes of the global Cartesian coordinate system, a coordinate value on the split axis and digital material definition in negative and positive coordinate direction.

A.2.6.2 Operation – Gradient

The gradient operation creates a linear gradient varying between two digital materials along a certain direction with a certain gradient length. The operation requires a gradient direction corresponding to one

of the axes of the global Cartesian coordinate system, coordinate values on the gradient axis to start and stop the gradient and digital material definition at the start and end of the digital material gradient.

A.2.6.3 Operation – Stencil

The stencil operation fills a space within the existing coarse grid with a digital material. This space can be defined by a set of coordinates provided as an $[n \times 3]$ matrix, where n is the number of coordinates and the columns correspond to the *x*-, *y*- and *z*-values, or by an STL file, where the filled space is the set of all coarse grid points within the STL geometry. The stencil operation is a two-step operation. First, the coarse grid points within the provided stencil space are assigned to the provided digital material. The second step is optional. It detects the edges of the stencil and blends the digital material of the base geometry and the digital material of the stencil over a user-defined number of coarse grid points. This is performed by a series of kernel operations detecting the edges and linearly smoothing the digital material values over the edge. Using the stencil operation including digital material smoothing enables the direct creation of smooth digital material transitions. A visualization of this procedure is shown in Supplementary Figure 7.

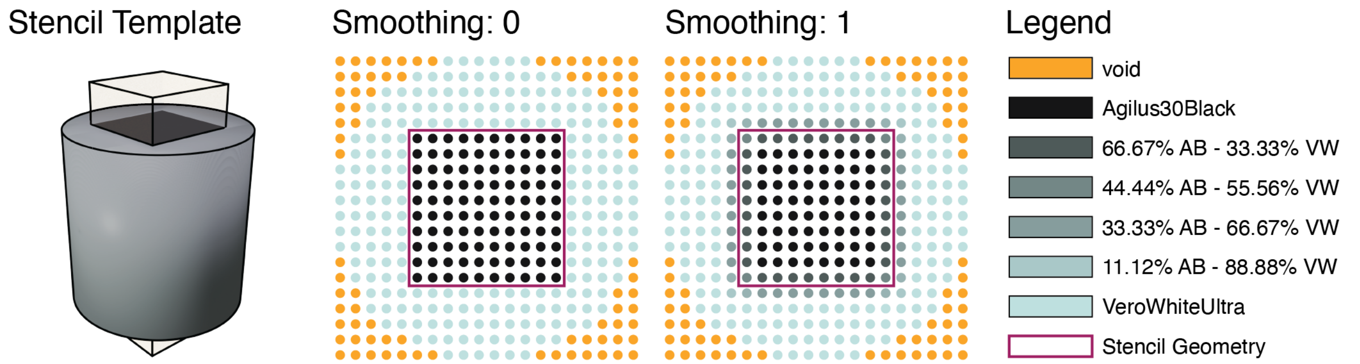

Supplementary Figure 7 Example of the stencil operation. From left to right; schematic of the stencil and part geometry; coarse grid layer after the stencil operation with no smoothing; coarse grid layer after the stencil operation with smoothing value one; digital materials and their mixtures in the coarse grid after applying the stencil with smoothing.

A.2.6.4 Operation – Coat

The coat operation detects the outer surface of the base geometry and coats it with a target digital material. The operation requires a coating thickness as well as coating digital material and core digital material. It creates a digital material gradient from the outside to the inside coat boundary if two different digital materials are provided.

A.2.6.5 Operation – Support

The support operation places a target digital material stated by the user to all void entries, which have a digital material entry in print direction anywhere above them. This operation is typically used as a last operation. It enables the placement of support only where it is needed to support the additively manufactured part instead of filling the entire bounding box with support material.

A.2.7 C – Master Curve Estimation

Prior to determining the Prony series for each AB/VW digital material in the coarse grid, several metrics providing input for the fitting procedure of the material model are derived. For the fitting procedure, the shift factor C_3, the glass transition temperature T_g, the longterm modulus k_0 and the master curve at room temperature are evaluated. The shift factor and the glass transition temperature are percolated with respect to the values of the two constitutive base materials Agilus30Black and VeroUltraWhite (Supplementary Figure 8). The master curve cannot be directly interpolated due to the high nonlinearity, induced by the temperature- and frequency-dependent percolation effect. It is estimated by an ANN with multi-layer perceptron architecture that is trained on a dataset of tuples of temperature, time scale, material mixture and stiffness data. It is set up to take three inputs; temperature, time scale and digital material

mixture value and estimate the corresponding digital material stiffness. The master curve for the digital material is thus assembled by the estimations of the ANN for a set of inputs having a constant digital material mixture value, a constant temperature equal to the room temperature of 22°C and a varying time scale. The time input is uniformly spaced on a logarithmic scale from $10^{-15}s$ to $10^{15}s$ with a discretization of 1000 points. The hyperelastic curve for the sample in the rubbery state (above T_g) is not used to fit the Prony series but is necessary to adapt the Prony elements for high strain values during simulation. It is interpolated between the data of AB and VW (Supplementary Figure 8).

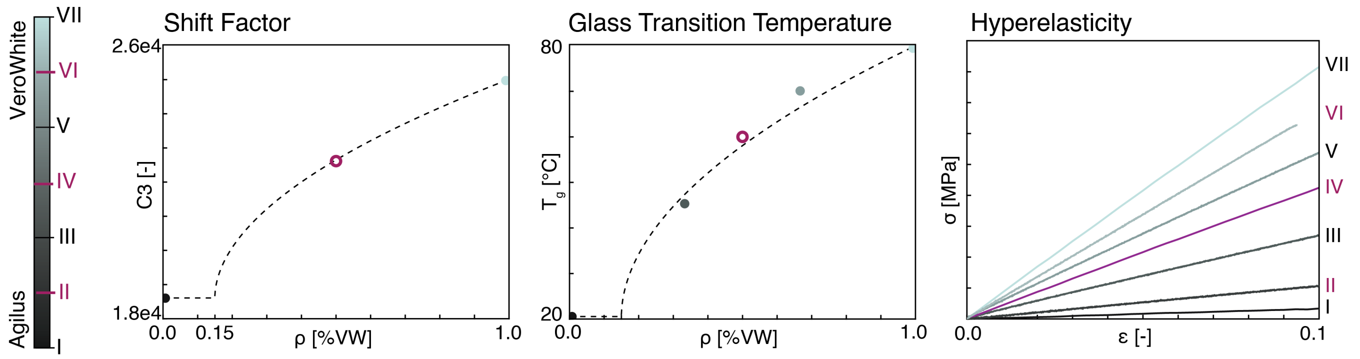

Supplementary Figure 8 Determination of coefficients for a target digital material. The shift factor and glass transition temperature are interpolated by percolation. The hyperelastic curve is interpolated between the data for Agilus30Black and VeroUltraWhite. Source data are provided as a Source Data file.

A.2.8 D – Prony Series Fitting Procedure

The fitting procedure for the estimated master curves is identical to the original fitting conducted on the master curves of the experimental results shown in Section A.1.2.

A.2.9 E – FE Mesh Generation

The FE mesh can either be generated automatically or given as a user input. If the mesh is provided as user input, it is provided as nodes and elements. The nodes are represented as a $[n \times 3]$ matrix, where n is the number of nodes, and the columns are the point coordinates in *x*, *y* and *z*. The elements are represented as a $[m \times 4]$ matrix, where m is the number of elements and the columns are the node IDs of the corner points of the tetrahedral elements. The automatic mesh generation differentiates in generating a mesh for *solid* and *shell* type geometries. *Solid* type geometries are approximated with a tetrahedral mesh using MATLABs inbuilt function `generateMesh` using linear elements. *Shell* type geometries directly output the already created triangular approximation as described in Section A.2.3. In all cases, the individual elements are assigned a digital material and sorted into material sets. The digital material assignment is based on the nearest coarse grid point to the element's centroid. It is thus important to note that gradients in the coarse grid on a smaller length scale than the FE mesh size cannot be depicted in the simulation.

A.2.10 F – PNG Creation

The PNG creation is performed individually for each print layer. The number of print layers is calculated from the parts bounding box height and the layer height h_{layer}. For each layer, five steps are performed: pixel grid creation, digital material interpolation, boundary determination, support material assignment and stochastic base material assignment.

During the pixel grid creation, a 2D grid with the provided *xy*-resolution and size according to the geometry's bounding box is created at the *z*-coordinate of the current layer. During digital material interpolation, all points in the newly created 3D grid are assigned a digital material based on linear interpolation from the digital materials in the coarse grid. During boundary determination, all points of the fine 2D grid are assigned to be either inside or outside the additively manufactured part to accurately additively manufacture the target geometry and not lose details due to aliasing by the coarse grid sampling.

This is performed using the same raytracing-based algorithm described in Section 0 on the provided STL part for *solid* type geometries and on the solidified triangular mesh according to Section A.2.3 for *shell* type geometries. During support material assignment, all points outside the geometry are either assigned void material or support material. A voxel is assigned support material if the interpolated digital material shows any percentage of designated support material, as stated by the function “Support” and assigned void otherwise. Finally, the stochastic assignment of the base materials is performed by using the base material percentages provided by the digital material for each point on the pixel grid.

A.3 Neural Network Training

In the proposed inverse design framework, two artificial neural networks (ANN) are utilized. One is used to estimate the AB/VW mixture ratio (ANN_{mix}) from provided tuples of target temperature, time scale and stiffness. The other is used to estimate the stiffness (ANN_E) for a given mixture ratio, temperature and time scale. Both ANNs are trained on a dataset generated from the proposed temperature- and frequency dependent percolation model. It consists of tuples containing temperature, time scale, mixture ratio and stiffness. The dataset is generated by calculating the digital material stiffness for each combination of temperature, mixture ratio and time scale using the previously fit percolation model. The temperature is sampled between $-30°C$ and $130°C$ with a step size of $1°C$, the mixture ratio is sampled between 0% and 100% AB content with a step size of 1% and the time scale is sampled between $10^{-15}s$ and $10^{15}s$. The time scale is sampled in the exponent to have 300 points with the exponent varying from –15 to 15. The data set thus consists of 4.8783 million data points. For training ANN_E, the full dataset is used. For training ANN_{mix}, the dataset is culled by removing all entries with a base material mix ratio above the percolation threshold of 16.3% AB. This is to prevent the same stiffness-time-temperature tuple having to be mapped to different mix ratios. This yields a dataset with 4.1055 million data points. Both, the full dataset and the culled dataset, are individually normalized to a scale between 0 and 1. The data for temperature, mixture ratio and stiffness is scaled linearly. The data for the time scale is first $\log 10$-transformed and then scaled between 0 and 1 in the logarithmic space. The normalization is reverted when using the neural networks in the design pipeline to have the unscaled values as the output.

The normalized datasets are randomly split into training and test data with a ratio of 80/20. The neural networks are both trained on the training data set and evaluated on the test data set. The networks ANN_{mix} and ANN_E are trained by taking the mixture ratio and stiffness as labels respectively and taking the remaining three variables as inputs. Both ANNs follow the same general architecture parametrized by the depth d, width w and activation function $a.$ They are fully connected multi-layer perceptrons having an input layer with three neurons, $d-2$ hidden layers of width w, and one output layer with one output neuron. Each neuron in the input and hidden layers has an activation function of type $a.$ The training of the ANNs is performed in Python 3.11.5 using PyTorch 2.2.0. The training is performed on the training data over n_e epochs using batchwise training with batch size n_b. The training is performed using the ADAM optimization scheme[6] using a custom loss function with learning rate lr. The loss function used to train ANN_{mix} is the mean squared error (MSE) between the estimated outputs and labels. The loss function used to train ANN_E is a weighted MSE plus a penalty function for negative stiffness estimations. It follows Supplementary Equation (4), where H is the Heavyside function, $\hat{y}$ is the ANNs estimate and y is the data label.

$$loss = \left[\frac{1}{n_b}\sum(\hat{y}-y)^2 \cdot \left(E_{range} \cdot (1-y)\right)\right] + w_{neg} \cdot \left[-\frac{1}{n_b}\sum H(-\hat{y})\right] \tag{4}$$

The weight w_{neg} is fixed to ten after initial empirical testing and E_{range} is set to 2096.7. The remaining hyperparameters d, w, a, lr, n_e and n_b are evaluated for both ANNs using a fully factorial hyperparameter tuning procedure. A set of values is defined for every hyperparameter based on preliminary empirical performance tests. The hyperparameter set is seen in Supplementary Table 3. A full-factorial training run using all combinations of the hyperparameters is performed on a high-performance computing cluster in parallel with 4GB of memory allocated for each job. The training duration with those settings depends on the hyperparameters and ranges between 2h and 12h. For each training run, the average training and test loss per batch in the last epoch is collected and the test loss is compared in a statistical analysis. During analysis, the hyperparameters are procedurally and heuristically fixed based on their interaction effects. Specifically, a hyperparameter is fixed to the lowest loss value whenever the interaction plot does not show interaction effects with other parameters. The interaction plot is then re-evaluated iteratively until the statistically best performing ANN is found.

In Supplementary Figure 9, the statistical hyperparameter analysis for the training of ANN_E is shown. In the first stage, the learning rate is fixed to 0.0001 since the interaction plot shows no or negligible interaction with any other parameters. In the second stage, depth, batchsize and the number of epochs is fixed to 7, 256 and 150 respectively for the same reason. The interaction plot for the width and activation

function, shows the best performing ANN to have a width of 150 and GELU activation function. The convergence of the training of the best performing network with $d = 7$, $w = 150$, $a = GELU$, $lr = 0.0001$, $n_e = 150$ and $n_b = 256$ is shown in Supplementary Figure 9d). Both the training and test loss converge to a low value and no overfitting is visible.

Supplementary Table 3 Hyperparameter set used during the hyperparameter evaluation.

Parameter	Values
Width	{30, 60, 90, 120, 150, 180}
Depth	{3, 5, 7}
Learning Rate	{1e-2, 1e-3, 1e-4}
Batchsize	{256, 512, 1024}
Activation Function	{GELU, ReLU, SELU}
Number of Epochs	{30, 60, 90, 120, 150}

In Supplementary Figure 10, the statistical hyperparameter analysis for the training of ANN_{mix} is shown. In the first stage, the learning rate is fixed to 0.001 since the interaction plot shows no or negligible interaction with any other parameters. In the second stage, the batch size and the number of epochs is fixed to 256 and 150 respectively for the same reason. Finally, the interaction plot for the width, depth and activation function shows a statistically good performance for the ANN that has a width of 180, a depth of 7 and GELU activation functions. The convergence of the training of the best performing network with $d = 7$, $w = 180$, $a = GELU$, $lr = 0.001$, $n_e = 150$ and $n_b = 256$ is shown in Supplementary Figure 10d). Both the training and test loss converge to a low value and no overfitting is visible.

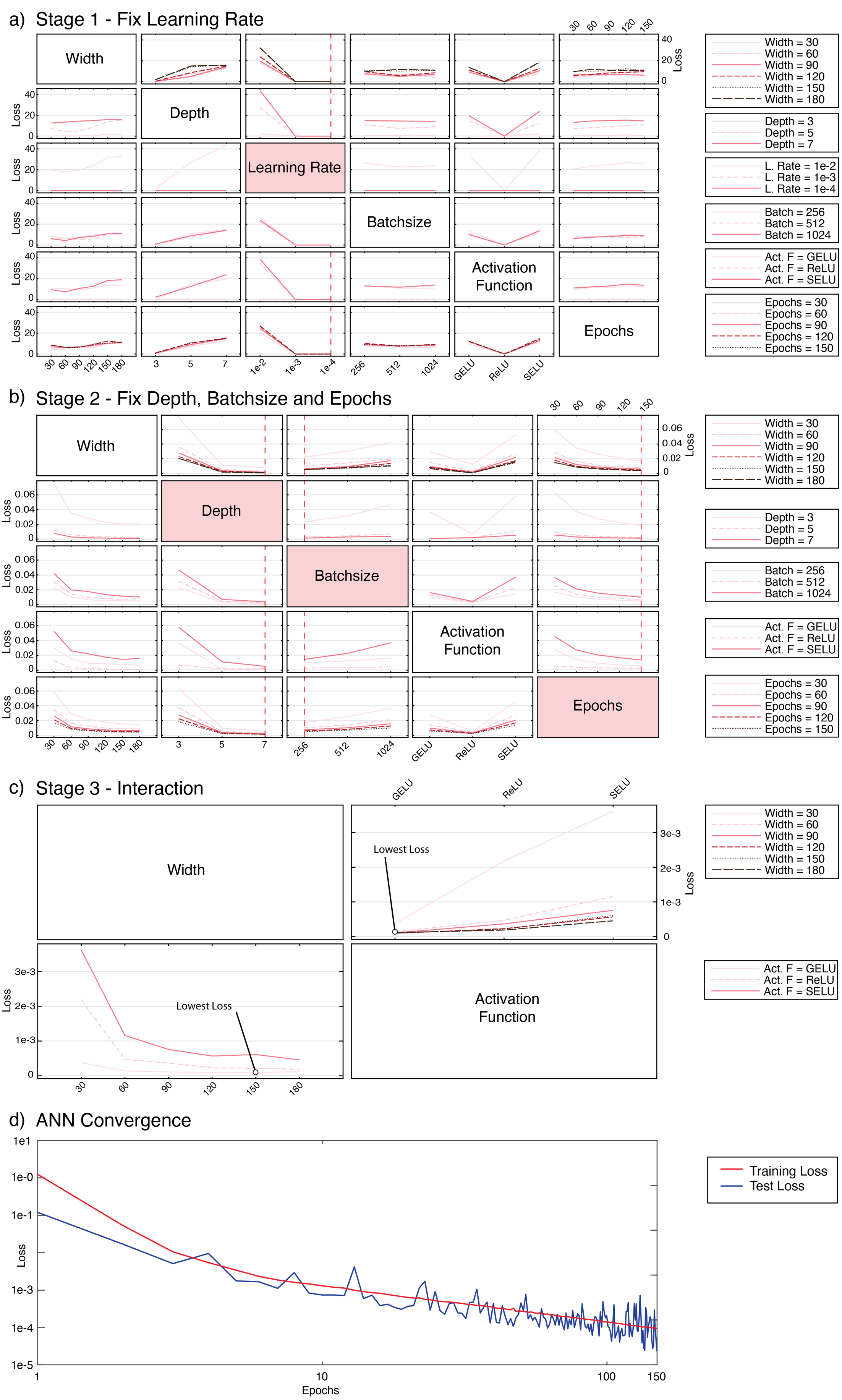

Supplementary Figure 9 Hyperparameter evaluation of ANN_{E} (a) Full interaction plot for all hyperparameters. The learning rate is fixed due to negligible interaction effects. (b) Interaction plot for the hyperparameter set reduced by the learning rate. The depth, batchsize and epoch number are fixed due to negligible interaction effects. (c) Interaction plot for the width and activation function. The lowest loss is found for a width of 150 and a GELU activation function. (d) Convergence of the training and test loss over all epochs for the best performing ANN. Source data are provided as a Source Data file.

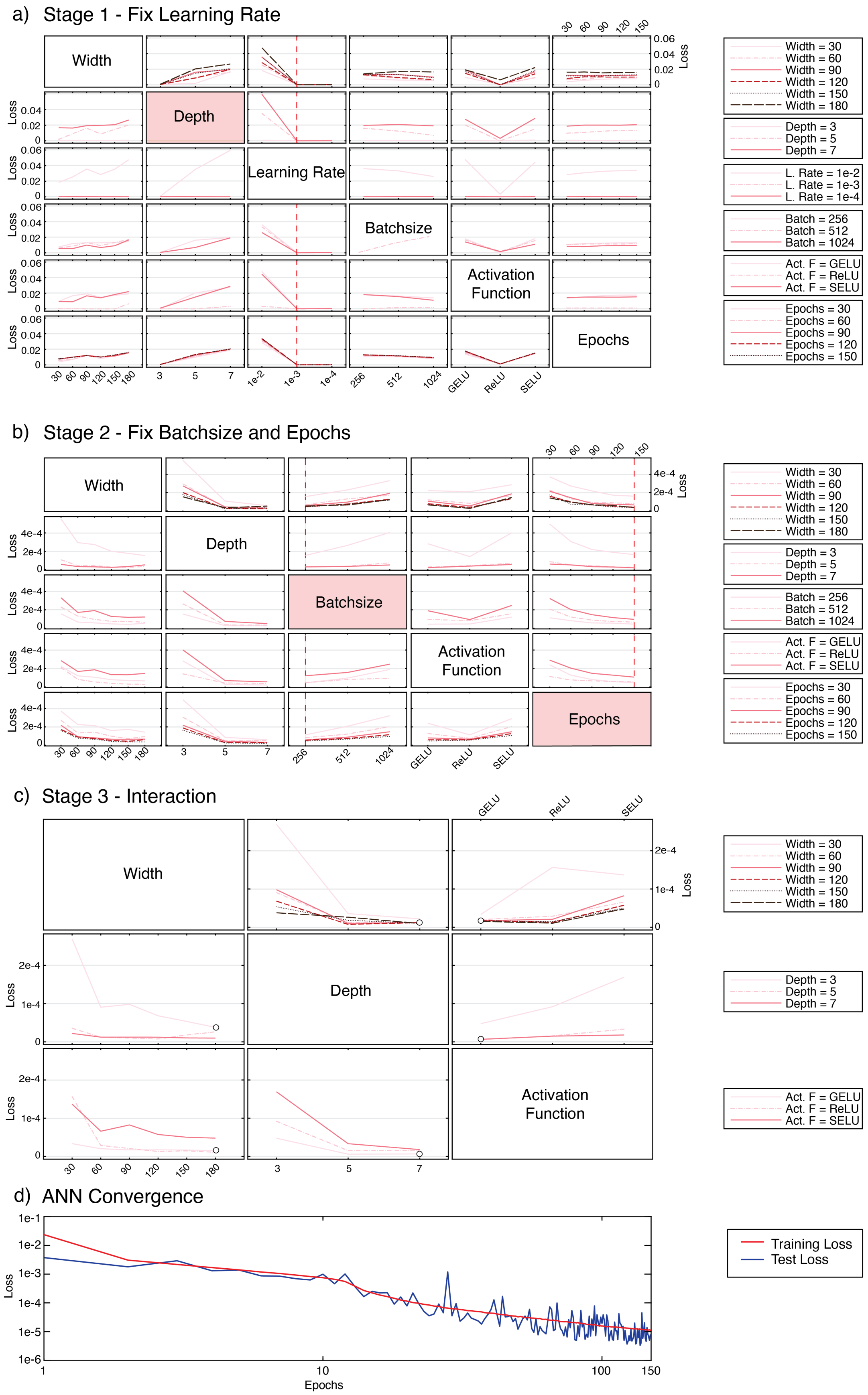

Supplementary Figure 10 Hyperparameter evaluation of ANN_{mix} (a) Full interaction plot for all hyperparameters. The learning rate is fixed due to negligible interaction effects. (b) Interaction plot for the hyperparameter set reduced by the learning rate. The batch size and epoch number are fixed due to negligible interaction effects. (c) Interaction plot for the width, depth and activation function. A good performing ANN is found for a width of 180, a depth seven and GELU activation functions. (d) Convergence of the training and test loss over all epochs for the best performing ANN. Source data are provided as a Source Data file.

A.4 Orthosis Details

A.4.1 Orthosis Requirements

The orthosis type chosen in this work is a static wrist orthosis, also called a wrist splint. Such splints are typically worn to treat carpal tunnel syndrome and osteoarthritis. It is crucial that they fit the patient's anthropometry for the best rehabilitation effect[7]. The main function of such a wrist orthosis is wrist immobilization. However, if possible, the hand's function should still be preserved[8] and the orthosis should protect the wrist from impacts during use to prevent further injuries[9]. Based on these design principles, four design requirements are derived. The orthosis must prevent flexion and extension of the wrist, adapt to long-term changes in the anthropometry, allow free thumb motion and be resistant to impacts. The design requirements are quantified in the following sections. For all derivations, a homogenous orthosis thickness of 3mm is assumed based on previous orthosis designs[9,10].

A.4.1.1 Wrist Immobilization

Research about wrist strength is mainly focused on healthy individuals[11,12]. In orthotics design, it is suggested to scale the maximum force exerted by healthy individuals since patients with injuries are either not capable of or do not want to exert a high force. Cazon et al.[10] propose scaling the force by a factor of 0.08, which translates to a wrist flexion and extension force of 14.9N and 8.4N respectively. For this force exertion, the orthosis must not deflect more than a certain amount. Zhou *et al.*[8] present an orthosis deflecting by 1.7mm and 2.2mm in flexion and extension respectively. Based on this work, the requirement for the wrist orthosis in this work is to not deflect more than 2mm for an applied force of 15N in both flexion and extension.

A.4.1.2 Orthosis Compliance

Orthosis compliance to swelling is assumed to be analogous to a reduction in material stiffness. There is a complex interplay between material deformation, material viscoelasticity and skin pressure and it is dependent on the orthosis and wrist geometry. For simplification, it is assumed that the orthosis does not deform significantly, which leads to the direct correlation of skin pressure and material stiffness. Physiological skin pressure is assumed to be below 25mmHg according to Kao *et al.*[13]. Pathologic skin pressure is mostly defined as external pressure above the capillary closing pressure of 32mmHg[14]. However, the harm due to pressure application increases significantly for increasing pressure, leading to deep tissue destruction at an external pressure of 50mmHg[15]. Since this work does not consider the interplay of deformation and pressure, a safety factor of three is applied. The orthosis must be capable of reducing a pathological skin pressure of 150mmHg to 25mmHg within one day.

A.4.1.3 Impact Resistance

The impact resistance is calculated based on physiological arm motion. Assuming an average body weight of 80kg, the weight of one arm is about 3% of the total body weight[16], yielding 2.4kg. The arm swing magnitude is 0.4m[17]. With a walking frequency of 2Hz[18] and assuming the speed of the hand as homogenous during the gait cycle, a hand travel speed of 0.4m/s is found. Together with the arms weight and a multiplication factor of 1.25 to consider any uncertainties, this yields an impulse of 1.2Ns when hitting external objects during everyday activities. Assuming an impact time of 0.01s, an impact force of 120N is calculated. The typical area of impact is assumed to be 1cm^2.

A.4.2 Orthosis Design

The orthosis is designed in Blender based on a publicly available 3D model of a hand and forearm[19]. The 3D hand model is smoothed manually, offset by 2mm and cut to have openings for the forearm and the fingers (Supplementary Figure 11). The shell is remeshed to get bigger faces and the three openings are cleaned to achieve a topology with equally spaced vertices. The stencils for the cutout of the thumb and the stiff top splint are modelled as 3D geometries such that the intersect between the shell and the cutout results in the target geometries. The cutout for the stiff splint is designed to not hit the bony landmark of the ulnar head and not be slimmer than 10mm. The width estimation is based on a bending cantilever model, the assumption is that only the splint with a stiffness of 2000MPa resists flexion and extension. The shell with cutouts, the splint and the thumb part are shown in Supplementary Figure 11.

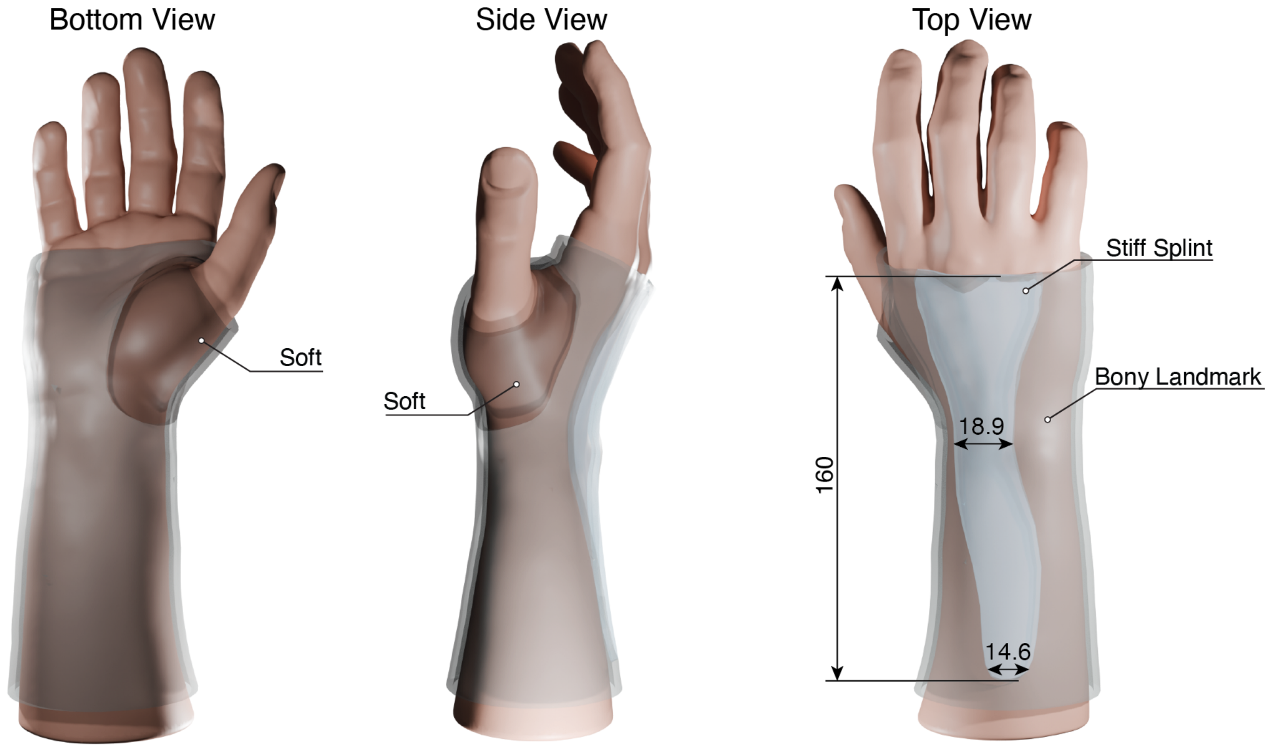

Supplementary Figure 11 Design of the wrist orthosis parts in bottom, side and top view. The shell is designed based on a 3D hand model. The stiff splint is designed to go around the ulnar head and have a minimum width of 10mm. The thumb region is designed using soft material to allow thumb motion. All images of the wrist are derived from a 3D scan provided by Theebadge/printables.com.

The orthosis is processed in MATLAB with the proposed framework[3] for voxel structure generation. The shell geometry is provided as a 2D *shell* type STL file and assigned a base material. The stiff splint and the soft thumb geometry are generated using the 3D stencils by applying the *stencil* operation. The coarse grid resolution is set at 4.1mm and both stencil operations are set to a blending value of one, yielding a linear transition of 8.2mm between the shell and the stencil parts. A total of 19 digital materials are generated to depict the digital material gradients. The digital material for the shell, the splint and the thumb are generated by providing mechanical requirements in the material assignment step. The thumb and the splint are required to be as soft and stiff as possible at 25°C respectively. The voxel structure generation framework estimates a digital material with a base material mix of 15.5/84.5% AB/VW for the thumb and 100% VW for the splint. The shell material is assigned a set of three requirement tuples to conform to both the impact and compliance requirements. The impact stiffness is evaluated by approximating the orthosis shell as a cantilever under three-point-bending. The cantilever is assumed to have 45mm side length to cover the worst-case scenario of very flat orthosis parts, e.g., at the palm. It has a thickness of 3mm and a maximum deflection of 2mm under a force application of 120N. This yields a target stiffness of 1250MPa at a time scale of 0.01s. For orthosis compliance the target stiffness between the time scales 1s and 86400s is reduced by 67.6% such that the initial stiffness is three times the final stiffness. The values of 900MPa and 150MPa are chosen to depict this effect. The estimation of the voxel slicer using this set of requirements, all required to hold true at a temperature of 25°C, yields a mixture of 26.5% AB and 73.5% VW. A summary of the requirements and the estimated digital material can be found in Supplementary Table 4. The coarse grid with the assigned material is shown in Supplementary Figure 12. The orthosis generation is finalized with the *support* operation.

Supplementary Table 4 Material requirements for orthosis design and the estimated digital materials.

Part	Requirement	Metrics	AB Content	VW Content
Thumb	Soft material	[25°C, 1s, 0MPa]	84.5%	15.5%
Splint	Stiff material	[25°C, 1s, 2000MPa]	0%	100%
Shell	Impact Resistance	[25°C, 0.01s, 1250MPa]	26.5%	73.5%
Shell	Stiffness before Swelling	[25°C, 1s, 900MPa]		
Shell	Stiffness after Swelling	[25°C, 86400s, 150MPa]		

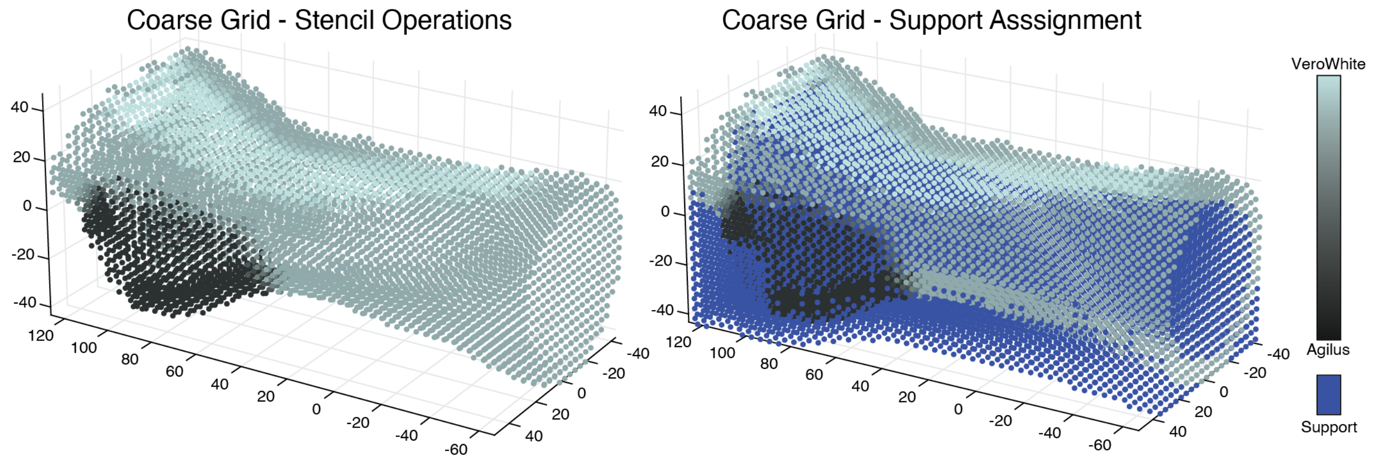

Supplementary Figure 12 Coarse grid with assigned digital material for the wrist orthosis. The orthosis material varies between a digital material mixed from 84.5% AB and 15.5% VW to a digital material mixed from 0% AB and 100% VW. SUP706 is assigned at 100% to the coarse grid points needed to support the structure during additive manufacturing.

A.4.3 Orthosis Simulation

A.4.3.1 Simulation Setup

The simulation of orthosis mechanics is performed in ABAQUS 2022. The mesh is automatically generated in MATLAB with a target element size of 4.1mm and contains 5587 linear triangular elements with reduced integration (S3R elements) and a thickness of 3mm. A total of 19 different digital materials mixed from AB and VW are assigned to the mesh varying in the AB content between 0 and 84.54%. The mesh and the digital material assignment are both shown in Supplementary Figure 13.

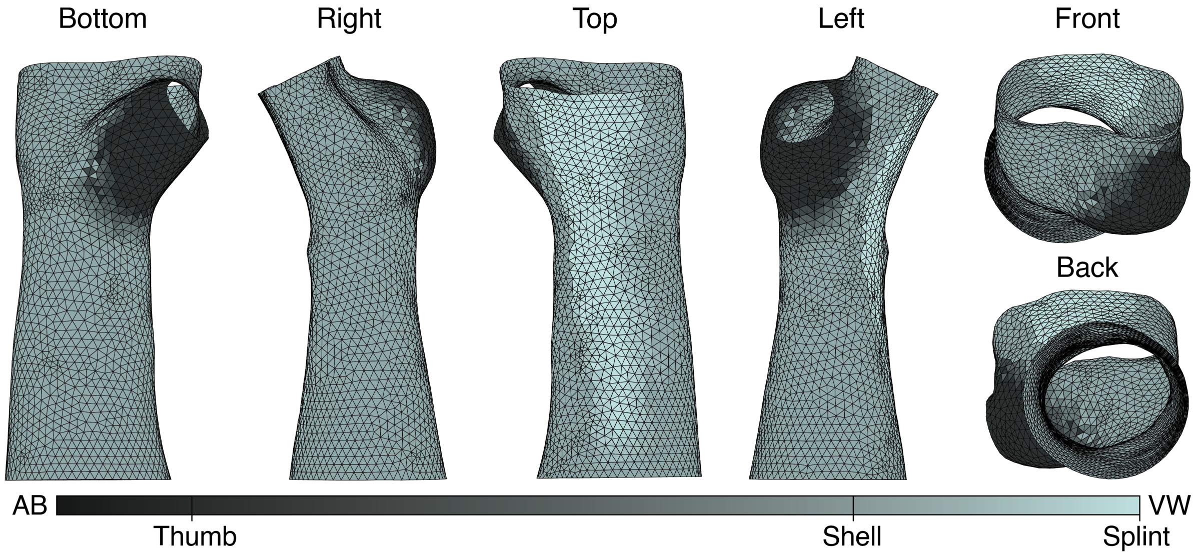

Supplementary Figure 13 Mesh of the wrist orthosis used for the FE simulations. The color indicates the digital material varying from Agilus30Black (dark) to VeroUltraWhite (bright). The elements have a target size of 4.1mm and are simulated as linear quadrilateral elements with reduced integration (S3R elements).

All simulations are performed by coupling node sets of the orthosis mesh to manually defined control points and applying constraints and loads to the control points only. The orthosis is thus split into three node sets corresponding to the forearm, the hand and the thumb. Further, a node set for the impact simulation is defined separately using a node group located on the side of the shell geometry. Each of these four node sets is assigned a control point with manually assigned coordinates in the global Cartesian coordinate system. A continuum distributing coupling with uniform weights is assigned between the node sets and the control points. A last control point is defined at the center of the thumb opening and assigned a kinematic coupling to all nodes at the edge of the thumb opening to prevent the soft material from collapsing during simulation. Lastly, a new cylindrical coordinate system is introduced at the location of the wrist joint. The cylindrical coordinate system is used to simulate angular wrist flexion and extension. The point sets, control points and the cylindrical coordinate systems are shown in Supplementary Figure 14. Simulations are conducted at $0℃$ (cold weather), $25℃$ (room temperature) and $37℃$ (body temperature).

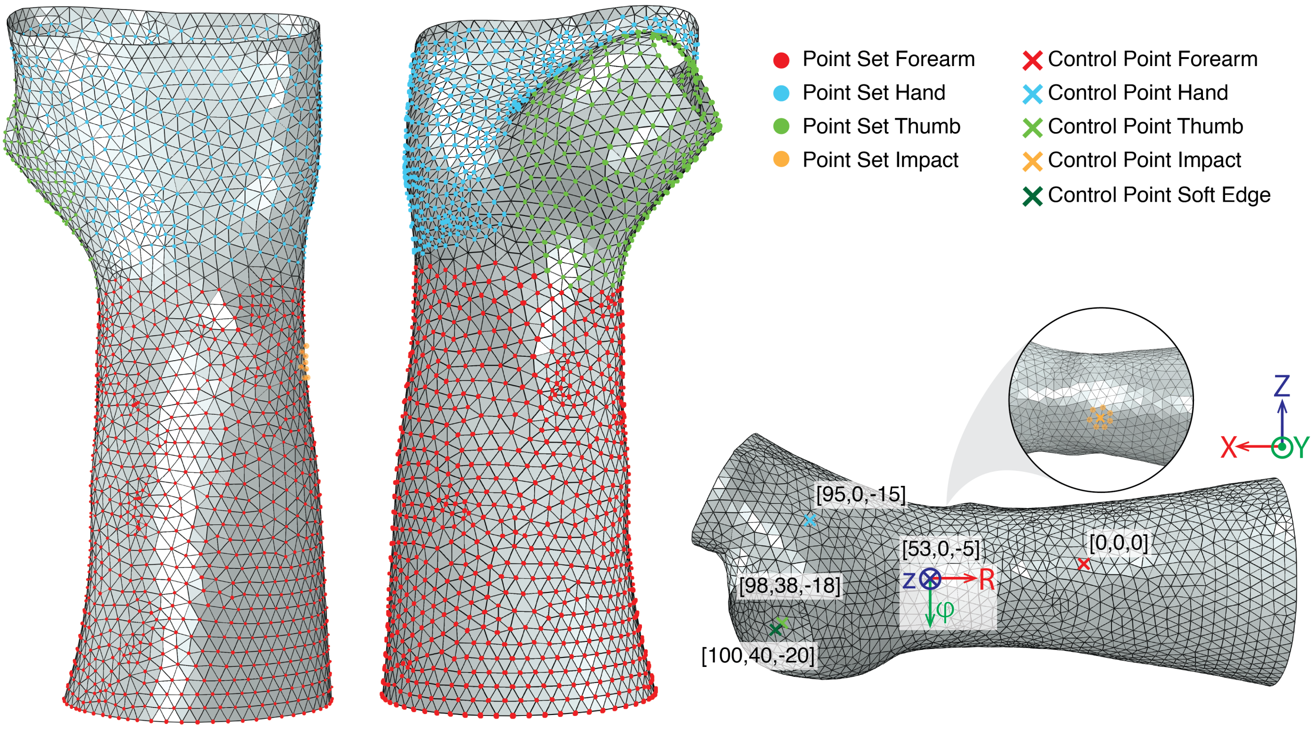

Supplementary Figure 14 Point sets and control points of the wrist orthosis in the FE simulation. The forearm, hand and thumb are all assigned to a mutually exclusive point set. The set for the impact simulation contains points belonging to the forearm. To prevent collapse of the soft thumb material, the edge of the thumb opening is kinematically constrained to a control point centered to the thumb opening. All other sets are constrained with a continuum distributing coupling to their respective control points.

A.4.3.2 Simulation Details – Wrist Flexion and Extension

Wrist flexion and extension is simulated by fixing the forearm control point and applying a force of 15N for flexion and –15N for extension to the hand control point along φ in the cylindrical coordinate system located at the wrist joint. The force is applied within 1s with a linear ramp. The coupling of the impact point set to the impact nodes is switched off. The initial increment is set to 0.01 with a minimum and maximum increment of 10^{-15} and 1 respectively. The viscoelastic strain error tolerance is set to 0.001.

The simulations both converge within a maximum of 35s on a single thread on an AMD Ryzen 9 7950X desktop CPU and 64GB of DDR-4 memory. A single thread is used as Abaqus 2022 exhibits problems with convergence in multithreaded viscoelastic simulations when using custom subroutines. The results for flexion and extension show a displacement between 0 and 1.24mm at the shell over all three simulated

temperatures (Supplementary Figure 30 and Supplementary Figure 31). The maximum von Mises stress over the shell thickness is between 0.481 and 0.813MPa for both flexion and extension, which is significantly below the tensile strength of AB and VW[20,21]. Simulation Details – Shell Compliance for Swelling

A.4.3.3 Simulation Details – Shell Compliance for Swelling

Orthosis compliance is simulated by fixing the forearm and hand control points, applying a pressure of 0.02MPa (150.01mmHg) to the forearm from the inside, fixing the resulting displacement and letting the orthosis relax for 86'400s. The coupling between the impact control point and the impact nodes is switched off. The force is applied within 1s with a linear ramp, the initial increment is of 0.01, a viscoelastic strain error tolerance of 0.001 and a minimum and maximum increment of $10^{-15}s$ and 1s are set. In the relaxation step, the initial increment is set to 0.01, the viscoelastic strain tolerance to 0.001 and the minimum and maximum increment to $10^{-15}s$ and $10^{4}s$ respectively.

The simulation converges within 95s on a single thread on an AMD Ryzen 9 7950X desktop CPU and 64GB of DDR-4 memory. A single thread is used as Abaqus 2022 exhibits problems with convergence in multithreaded viscoelastic simulations when using custom subroutines. The results show a decrease in the average von Mises stress over the shell thickness in the range of 26.4% ($0℃$) and 70.5% ($37℃$). The reduced amount of relaxation at $0℃$ would likely only be during short exposure times over the whole day, room temperature and body temperature are those the more prevalent temperature ranges for swelling. Stress concentrations are visible at the dorsal side of the wrist. This is since the pressure is only applied to the forearm. This transition from forearm to hand combined with the concave local geometry causes buckling at that region leading to higher stresses. However, the overall behavior of the shell material is not influenced by this.

A.4.3.4 Simulation Details – Impact

An impact on the orthosis is simulated by fixing the forearm and the hand control point and applying a force with magnitude 120N to the impact control point. The force is applied with an attack angle of 30° with respect to the global *y*-axis in the *yz*-plane. This corresponds to a force vector of $[0\ 104\ -60]\ N$ in the global coordinate system. The force is applied within 0.01s using a linear ramp. The initial increment is set to 0.0001s with a minimum and maximum increment of $10^{-15}s$ and $0.01s$ respectively. The viscoelastic strain error tolerance is set to 0.001. The simulation converges within a maximum of 33s in 12 on a single thread on an AMD Ryzen 9 7950X desktop CPU and 64GB of DDR-4 memory. A single thread is used as Abaqus 2022 exhibits problems with convergence in multithreaded viscoelastic simulations when using custom subroutines. The results show a maximum displacement of 0.0274mm and a maximum stress over the shell thickness of 0.103MPa, both appearing in the vicinity of the impact (Supplementary Figure 33). Even at body temperature the orthosis provides protection against impacts due to the low displacement magnitude and low local stress (compared to the tensile strength of AB and VW) levels during impact. Shell Material Compliance Test

Testing of the long-term compliance of the orthosis shell material is performed in a specialized setup using the Instron ElectroPuls E3000 (Instron, Norwood, MA, USA) tensile testing machine with an Instron Dynacell 2527 having a range of ±250N. The part under test is a cylindrical shell with a length of 50mm, an inner radius of 60mm and a thickness of 3mm thus approximating a typical simplified orthosis geometry. It is additively manufactured using the voxel slicer presented in this work with the digital material assigned to the orthosis shell in the curved geometry, VeroUltraWhite at clamped ends and a linear gradient between. The part under test is clamped between two additively manufactured parts manufactured using PETG on an E3D Motion System and ToolChanger (E3D, Chalgrove, United Kingdom) such that the top part has 0.5mm distance to the part under test. The clamped assembly is attached to a steel rod connected to the load cell on the tensile testing machine. An additively manufactured stamp with a radius of 60mm at the tip, also manufactured on the E3D ToolChanger using PETG, is attached analogously to the moving crosshead of the testing machine. The test is performed by applying a target force of 6N via the stamp to the part under test within 1s and hold the displacement after force application for 86'400s (one day). The test setup and the raw measurement data after force application is shown in Supplementary Figure 15. It

can be observed that there is an unexpected stiffness increase at the later stage of the measurement. This is most likely due to environmental temperature variations during the night.

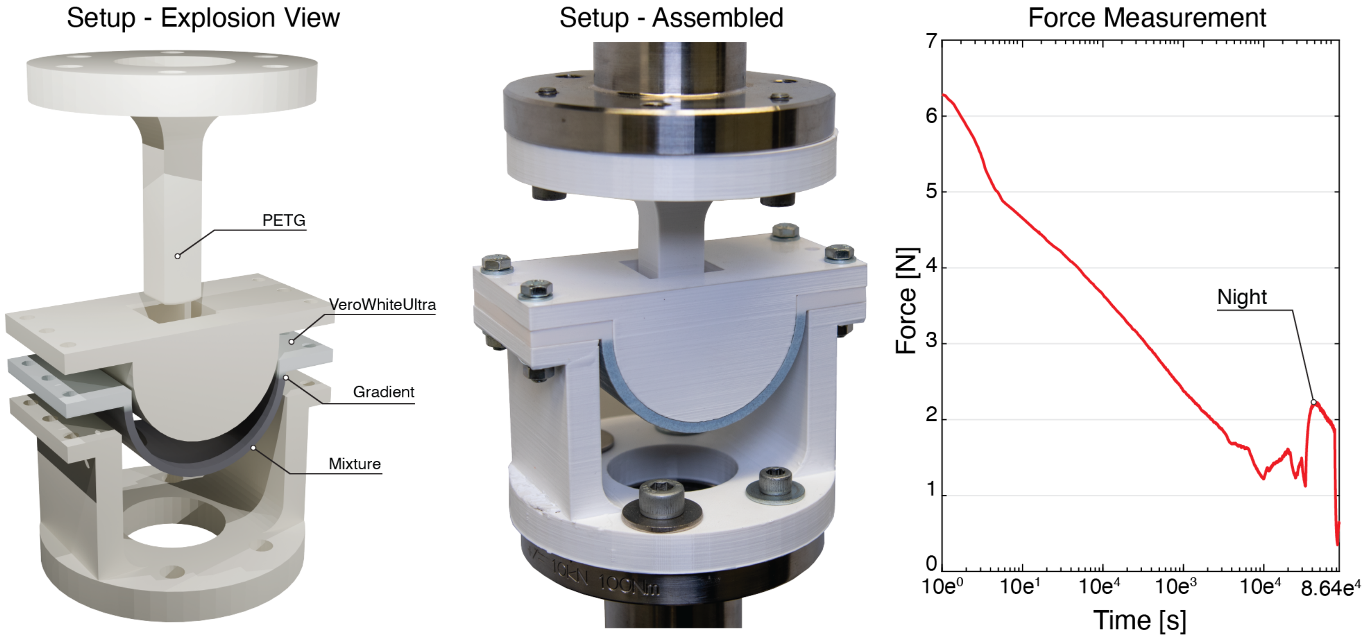

Supplementary Figure 15 Test setup of the relaxation test for the orthosis shell material. The setup consists of a test part with the digital material under test clamped between two parts out of PETG. A PETG stamp presses on the digital material with a set force and keeps the displacement for 24h. The measured force shows the decrease of the materials stiffness. Source data are provided as a Source Data file.

A.4.4 Orthosis Deformation Test

The flexion and extension of the wrist orthosis is tested by using a phantom of the wrist and applying a 1.5kg load to the phantom's hand. The phantom is shown in Supplementary Figure 34 on the left. It is designed from the same hand geometry as the wrist orthosis. The phantom is split into three parts: the forearm, the wrist joint and the hand. The forearm geometry corresponds accurately to the hand geometry used for orthosis design, while the hand geometry is processed to be inserted into the orthosis from the front. This is achieved by cutting parts of the palm and offsetting the total geometry slightly to the inside. The hand and the forearm phantom parts are additively manufactured using the E3D Motion System and ToolChanger system with PETG. The joint is additively manufactured on the J850 Prime using VeroUltraWhite. The joint is glued to the hand and connected, after donning the orthosis, with a screw to the forearm. The phantom/orthosis assembly is fixed to a base and a load of 1.5kg is applied at the hole in the phantom's hand. A photo is taken before and after load application. The photos are overlaid digitally, and the maximum deformation is evaluated visually and quantified using references in the photo. The maximum deformations are 1mm and 0.49mm for flexion and extension respectively, which is similar to the deformation during simulation. The observed difference could have been introduced by a variety of factors; the image-based measurement, the force application and the simulation setup. The image-based measurement is error prone since the projection is distorted by perspective, the references in the images might not be perfectly accurate, and overlaying the images can introduce errors. The force application is performed at the orthosis opening for the experimental validation while it is applied at the palms center in the simulation. This increases the lever arm relative to the wrist joint, increasing the effective load applied. Lastly, the simulation is set up by applying a force to the hand control point coupled to all points assigned to the hand point set. This is different to the experiment, where the orthosis is not directly touching the hand part opposite of the force direction, which leads to a more compliant behavior in the experiment. Considering all factors, the match of simulation and experiment is still acceptable, especially since all measurements are still 50% below the maximum deformation of 2mm.

A.5 Damper Details

A.5.1 Damper Design

The damper type chosen in this work is loosely based on a Vibrostop AAT 20/N damper (Vibrostop SRL, Milan, Italy). Such dampers are typically used for suspending pipes, ducting, speakers and ceilings. The use of a damper in the suspension of these components allows the interruption of vibration and noise transmission routes. The main function of such a damper is to dissipate vibrational energy through hysteresis and carry the weight of whichever component is attached to it. This specific damper is chosen due to its general design and more complicated interfaces between the rigid shell material and the inner damping material compared to more typical double bolt vibration isolators. It is also subject to a contact condition on the bottom surface making the expected hysteresis complex enough to benchmark the proposed material modeling approach.

The damper is processed in MATLAB with the proposed framework[3]. The damper geometry is provided as a 3D STL file and assigned a rigid base material (VW). The soft damper geometry is generated using a 3D stencil by applying the *stencil* operation with a digital material consisting of 83.5% AB and 16.5% VW. The coarse grid resolution is chosen to be 0.5mm and the stencil operation is set to a blending value of one, yielding a linear transition between the housing and the stencil parts of 1.0mm. With that, a total of 28 digital materials are generated to depict the digital material gradients. The digital materials for the housing and the damper are generated using the material assignments from the initial import and the *stencil* operation. The damper generation is finalized with the *support* operation. The coarse grid with the assigned digital materials and the version also containing support material are shown in Supplementary Figure 16.

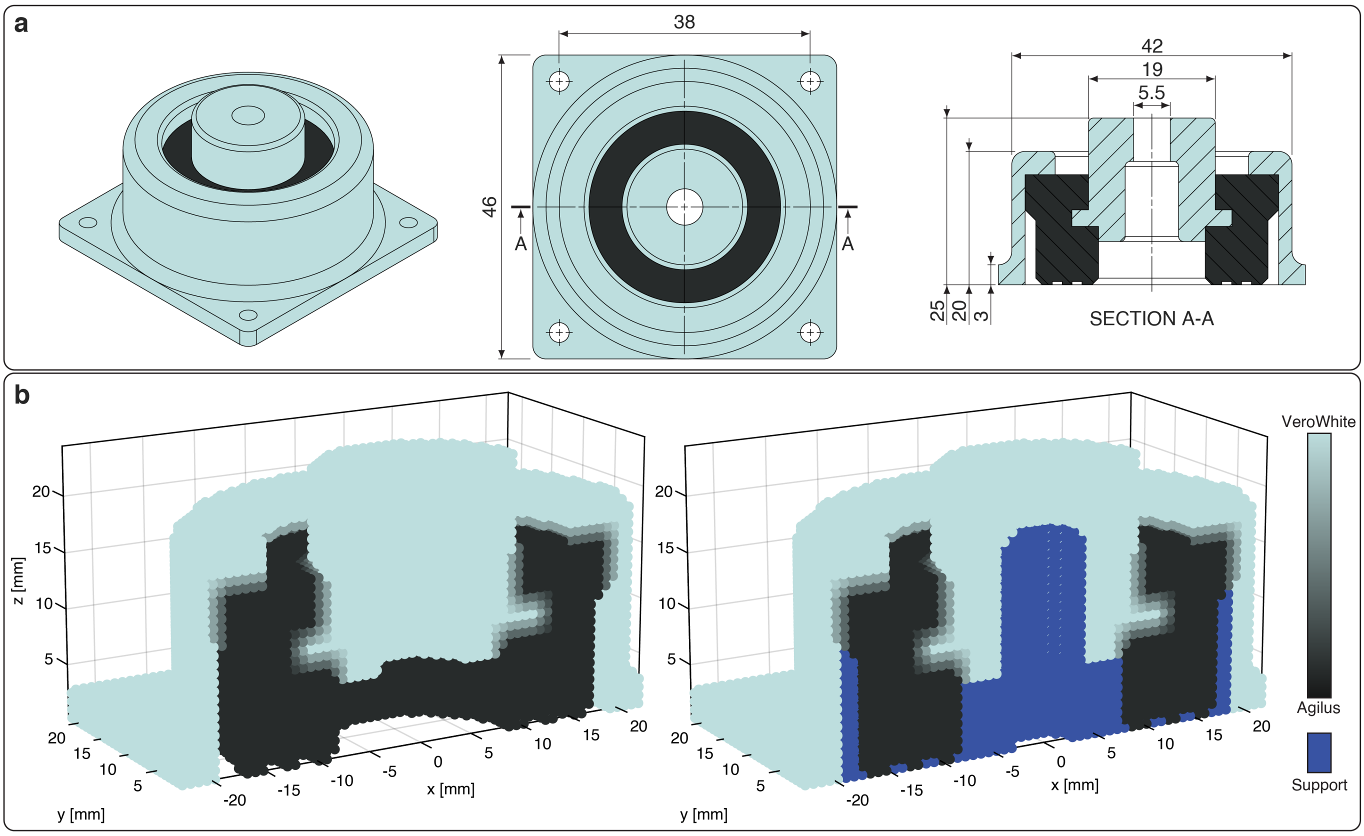

Supplementary Figure 16 (a) Technical specifications of the original machine damper design. (b) Coarse grid of the assigned digital materials for the voxelated damper. The damper material varies between a mixture of 83.5% AB and 16.5% VW to a mixture of 0% AB and 100% VW. SUP706 is assigned to the coarse grid points needed to support the structure during additive manufacturing.

A.5.2 Hysteresis Simulation

A.5.2.1 Simulation Setup

The simulation of the damper hysteresis under cyclic loading is performed in ABAQUS 2022. The mesh is generated in Abaqus to allow finer resolution meshing around the graded material regions. The target element size in homogenous material regions is 1.5mm, in the graded regions the target element size is 0.5mm. The mesh contains 253’777 linear tetrahedral elements with reduced integration (C3D4H elements). A total of 28 different AB/VW digital materials are assigned to the mesh varying in the AB content between 0% and 83.5%. The mesh and the digital material assignment are both shown in Supplementary Figure 17.

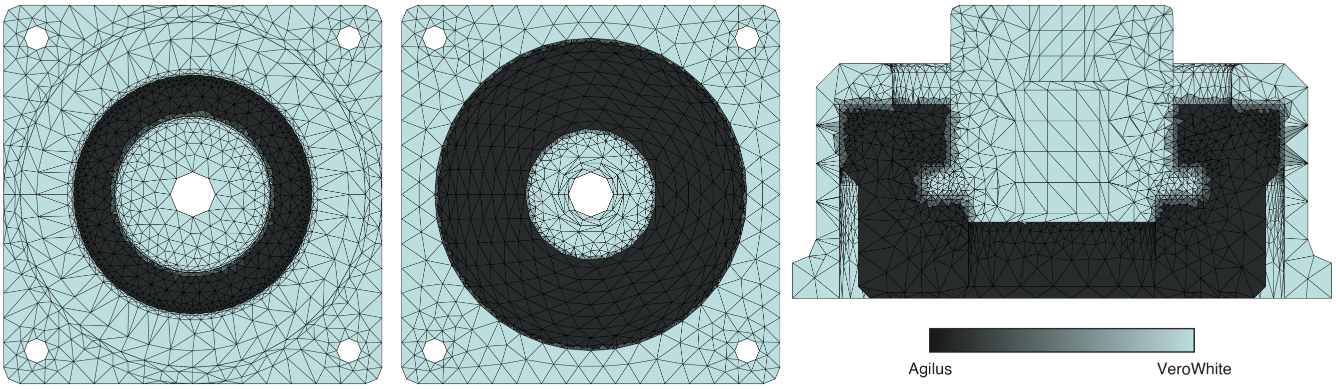

Supplementary Figure 17 Mesh of the damper used for the FE simulations. The color indicates the digital material varying from Agilus30Black (dark) to VeroUltraWhite (bright). The elements have a target size of 1mm in homogenous regions and 0.5mm in graded regions. The mesh consists of linear tetrahedral elements (C3D4H elements).

The simulation is performed by coupling node sets of the damper mesh to manually defined control points and applying constraints and loads to the control points as well as applying some constraints directly to the nodes. The loading at the top of the damper is applied using a continuum distributing coupling with uniform weights and a single control point (Supplementary Figure 18 in red) in both tension and compression. The bolts holding the housing in place are also modeled using continuum distributing couplings with uniform weights with a separate control point per bolt (Supplementary Figure 18 in green). Lastly, the bottom surface of the housing and damping material are gathered into one node set (Supplementary Figure 18 in blue). This node set is added to a frictionless contact interaction with a rigid plane placed onto the bottom surface.

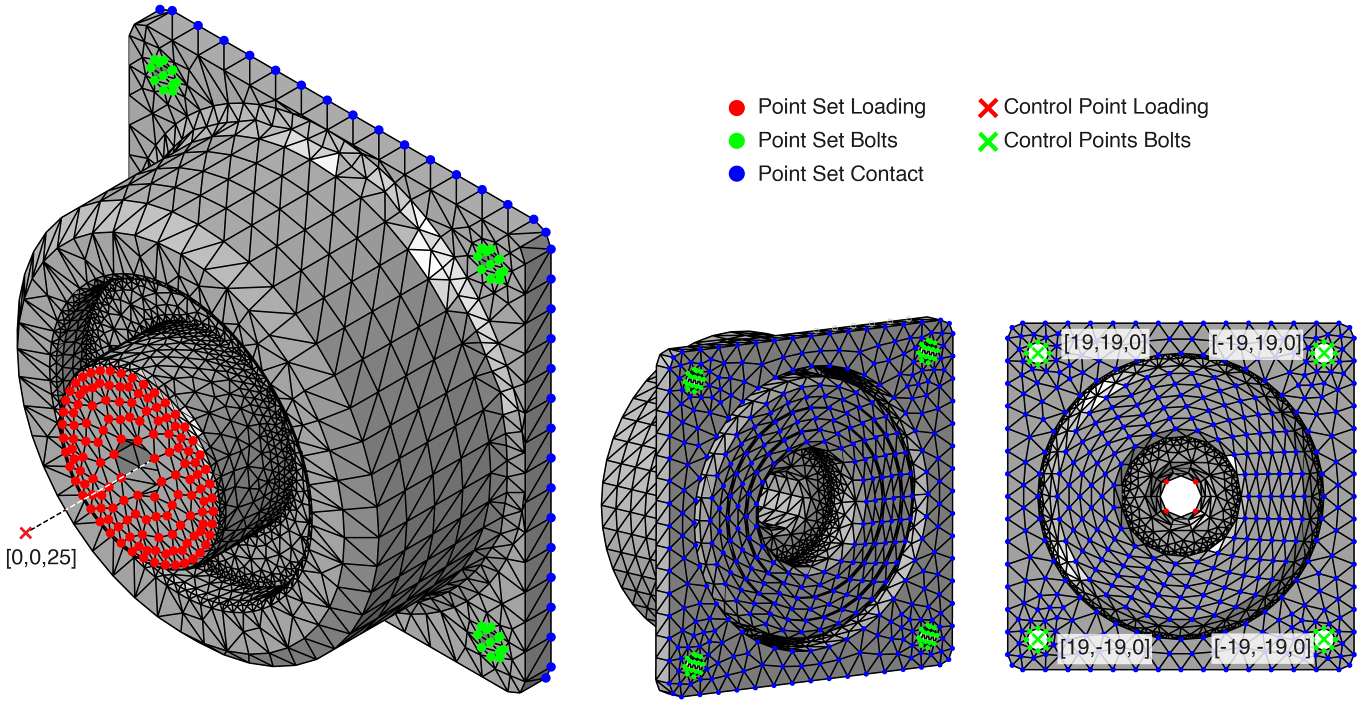

Supplementary Figure 18 Point sets and control points of the damper in the FE simulation. The bottom surface is modeled as a set of points to allow for the introduction of a frictionless contact condition. All other sets are constrained with a continuum distributing coupling to their respective control points.

A.5.2.2 Simulation Details – Hysteresis

The hysteresis of the damper is simulated by fixing the bolt control points in all three translational directions (*x, y, z*) and two out of three rotational directions, the only unfixed degree of freedom is rotation around the bolt's own axis. The simulation is displacement controlled and the initial unloaded damper is assumed to be at a zero position. Key simulation parameters are shown in Supplementary Table 5.

Supplementary Table 5 Damper simulation parameters.

Parameter	0.1Hz	1Hz	10Hz
Analysis Type	Static, Visco	Static, Visco	Static, Visco
NLGEOM	Enabled	Enabled	Enabled
Displacement Amplitude	0.5 mm	0.15 mm	0.15 mm
Strain Error Tolerance	0.001	0.001	0.001
Nodal Temperature	$\{15℃, 25℃\}$	$\{15℃, 25℃\}$	$\{15℃, 25℃\}$
Time Period	20s	2s	0.2s
Min Step Size	1E-15 s	1E-15 s	1E-15 s
Max Step Size	0.5 s	0.05 s	0.005 s

The displacements are applied using a sinusoidal amplitude to simulate vibrations at different frequencies. The simulation uses the ABAQUS VISCO step with nonlinear geometry modeling enabled. The simulation converges in between 100 and 145 minutes on a single thread on AMD Ryzen 9 7950X desktop CPU and 64GB of DDR-4 memory. A single thread is used as ABAQUS 2022 exhibits problems with convergence in multithreaded viscoelastic simulations when using custom subroutines. The experimental testing setup assumes that the transducer applying the displacement is at the top of the damper and the load cell is at the bottom. To model this accurately in the observed hysteresis behavior,

the displacement used to calculate the hysteresis is the displacement of the top surface of the damper. The reaction force is evaluated at the bottom surface contact and bolt control points. The first and second cycle of the cyclic load are simulated. An evaluation of the maximum displacements and von Mises stresses for all three frequencies at 15℃ can be found in Supplementary Figure 28 and Supplementary Figure 29.

A.5.3 Hysteresis Test

The experimental validation of the damper's hysteresis behavior is performed in a specialized setup using the Instron ElectroPuls E3000 (Instron, Norwood, MA, USA) tensile testing machine with an Instron Dynacell load cell having a range of ±5kN. The damper is fixed to an additively manufactured jig, the jig is manufactured using PETG on an E3D Motion System and ToolChanger (E3D, Chalgrove, United Kingdom). The top of the damper is fixed to an M4 bolt, this bolt is then clamped in a special wedge action tensile grip. The PETG jig at the bottom is also clamped into a tensile grip. The transducer at the top of the testing machine imposes the required sinusoidal displacement, the load cell at the bottom measures the corresponding reaction force. All data during testing is recorded using Instron WaveMatrix. The test setup is shown in Supplementary Figure 19.

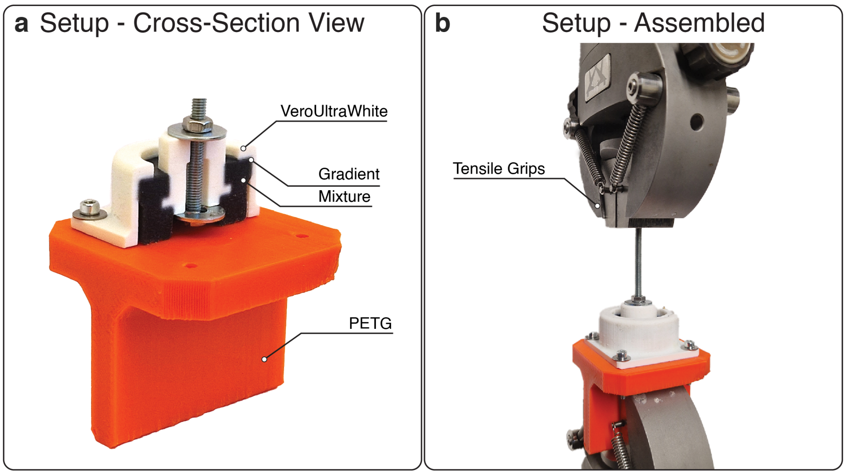

Supplementary Figure 19 (a) Cross-section of the mounting setup used to test the additively manufactured voxelated damper. (b)The fully assembled test setup on the Instron machine.

B. Supplementary Discussion

B.1 Runtime Evaluation

The framework's runtime performance is analyzed for the two case studies: the machine damper and the wrist orthosis. Supplementary Figure 20 shows the runtime of different parts of the framework according to Fig. S6 for both case studies. The runtime is provided for running the code on an Apple M3 Max Chip (Apple Silicon architecture) running macOS Sequoia 15.5. PNG creation and the digital material model fitting both contribute significantly to the total calculation time. Since both PNG creation and digital material model fitting are only performed once the user has found a suitable design, the time overhead is not considered important. It can be observed that the material assignment step is very fast, which shows the capability of iterating quickly over a large variety of material assignments until a satisfactory design is found.

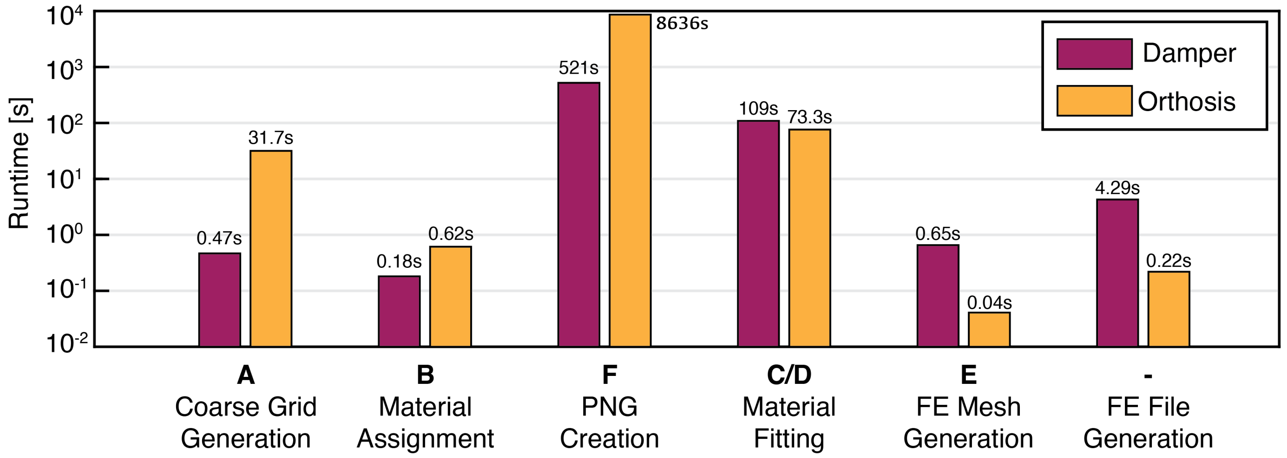

Supplementary Figure 20 Runtime of different parts of the voxel generation framework for the two presented case studies.

B.2 Assessment of Percolation Threshold

The stochastic mixing of two base materials, which constitute a digital material, is modeled using a modified version of the de Gennes percolation theory[22]. Percolation theory in this context describes how the homogenized digital material behaves based on the volume fractions of the individual base materials. As the VW volume fraction ρ_{vw} increases, it transitions from dispersed droplets to an interconnected network[23]. As shown in Supplementary Equation (5):

$$r = \frac{(\rho_{vw} - \rho_c)^{\mu}}{(1 - \rho_c)^{\mu}} \tag{5}$$

the effective mix ratio r in its normalized form is dependent on the percolation coefficient μ, the percolation threshold ρ_c and the VW volume fraction. The percolation threshold ρ_c is set estimated to be around 0.15 based on previous literature[24]. To qualitatively validate the percolation threshold of 0.15, a series of samples with different digital materials that have mixing rations between 0% AB and 30% AB are manufactured and visually inspected. Supplementary Figure 21a shows the manufactured samples, and Supplementary Figure 21b highlights the observed connected regions of AB. These images reveal a transition from interconnected AB domains to dispersed droplets between 15% and 20% AB content, supporting the literature-based threshold of around 0.15.

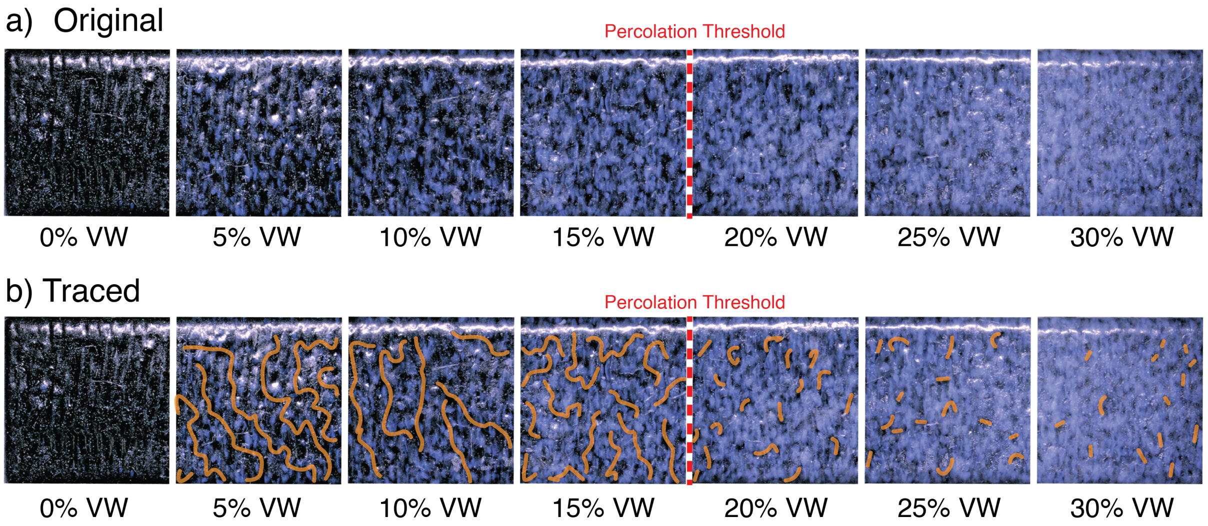

Supplementary Figure 21 (a) Close-up images of digital materials consisting of AB and VW with AB concentrations varying between 0% and 30% in 5% steps. (b) Visually traced connected AB regions.

The final percolation threshold is determined based on an additional sample from the frequency sweeps. Samples I, III, V and VII from Fig. 3 are used to determine the percolation coefficient μ, sample II is used to determine the percolation threshold ρ_c. This is achieved by minimizing the mean squared error of the storage modulus E' of the experimental master curve of sample II relative to the predicted percolated master curve generated from samples I, III, V and VII over the full frequency range of 10^{-15} to $10^{15} Hz$:

$$\min_{\rho_c} \left(E'_{exp} - E'_{perc}(\rho_c) \right)^2 \quad s.t. \quad 0 \le \rho_c \le 0.3 \tag{6}$$

According to this fitting procedure a percolation threshold of 0.163 is optimal at a temperature of $22\,°\mathrm{C}$. This temperature is chosen due to the subsequent fitting procedure (Section A.2.7) used to calculate arbitrary digital materials.

B.3 Design and Manufacturing Repeatability

The repeatability of the stochastic digital material is influenced by two sources of variation. The first is the process-related variability of the polymer material jetting system. The second source of variation is the stochastic nature of the voxel-level base material assignment. The jetting process introduces deviations due to factors such as print head dynamics, droplet formation, curing conditions and roller activity. Separately, the stochastic base material assignment process results in deviations from the target base material mix ratio. These two sources can independently contribute to a variation in the mechanical response.

Both sources are experimentally characterized using uniaxial tension tests on Type V dogbone specimens (ASTM D638) with a graded gauge section. As shown in Supplementary Figure 22a, the gauge transitions from 35% to 65% AB content. The ends consist of pure VW with a linear gradient to the gauge section. Each sample is tested at 3mm/min up to 2mm extension.

To assess the manufacturing variability, 21 samples are additively manufactured with the same stochastic seed. The voxel layout is thus identical. The average peak reaction force is 109.1N and the standard deviation is 3.2N. The manufacturing induced variability is thus 2.93% (Supplementary Figure 22b).

To assess the design-induced variability from stochastic mixing of base materials, another 20 samples are additively manufactured with different random seeds. They thus have different voxel layouts. As shown in Supplementary Figure 22c, the average peak reaction force is 108.5N and the standard deviation is 2.7N. This corresponds to a variability of 2.49%. This is lower than the manufacturing-induced variability.

This suggests that the stochasticity of the base material assignment has a minor impact on mechanical repeatability for samples of digital material at the tested scale.

The impact of stochastic base material assignment is also analyzed analytically. For a cube of digital material of a given feature size, the number of voxels n within the cube determines the effective sample size of the binomial mixing process. Assuming voxel-wise base material assignment with probability p, the standard deviation in the number of voxels allocated is calculated using Supplementary Equation (7).

$$\sigma_n = \sqrt{n \cdot p \cdot (1-p)} \tag{7}$$

Normalizing this by n yields the standard deviation in the base material mix ratio. Due to the nature of the binomial distribution, the error is maximal for $\rho = 0.5$. Supplementary Figure 22e shows the predicted standard deviation in ρ for feature sizes between 0.1mm and 10mm for the target mixture ratios of 0.1 and 0.5. For sub-millimeter features, the mismatch exceeds 1-10% while for features above 1mm, it remains below 1% and drops below 0.1% above 5mm.

The mismatch in base material mix ratio is mapped to maximum stiffness error by using the proposed material model across all temperatures and frequencies. This provides the maximum possible stiffness error band when assigning a target mixture for a digital material. As shown in Supplementary Figure 22f, the stiffness error becomes substantial for feature sizes below 1mm. Depending on the target digital material, the stochastic effects overshadow the manufacturing variability at feature sizes below 0.8mm and 1.8mm respectively. These findings confirm previous findings in literature that feature sizes below 2-5mm are highly sensitive to stochastic effects[25,26]. In conclusion, homogenization of voxelated digital material for this manufacturing process should be applied with a minimum feature size in the millimeter range to ensure accuracy.

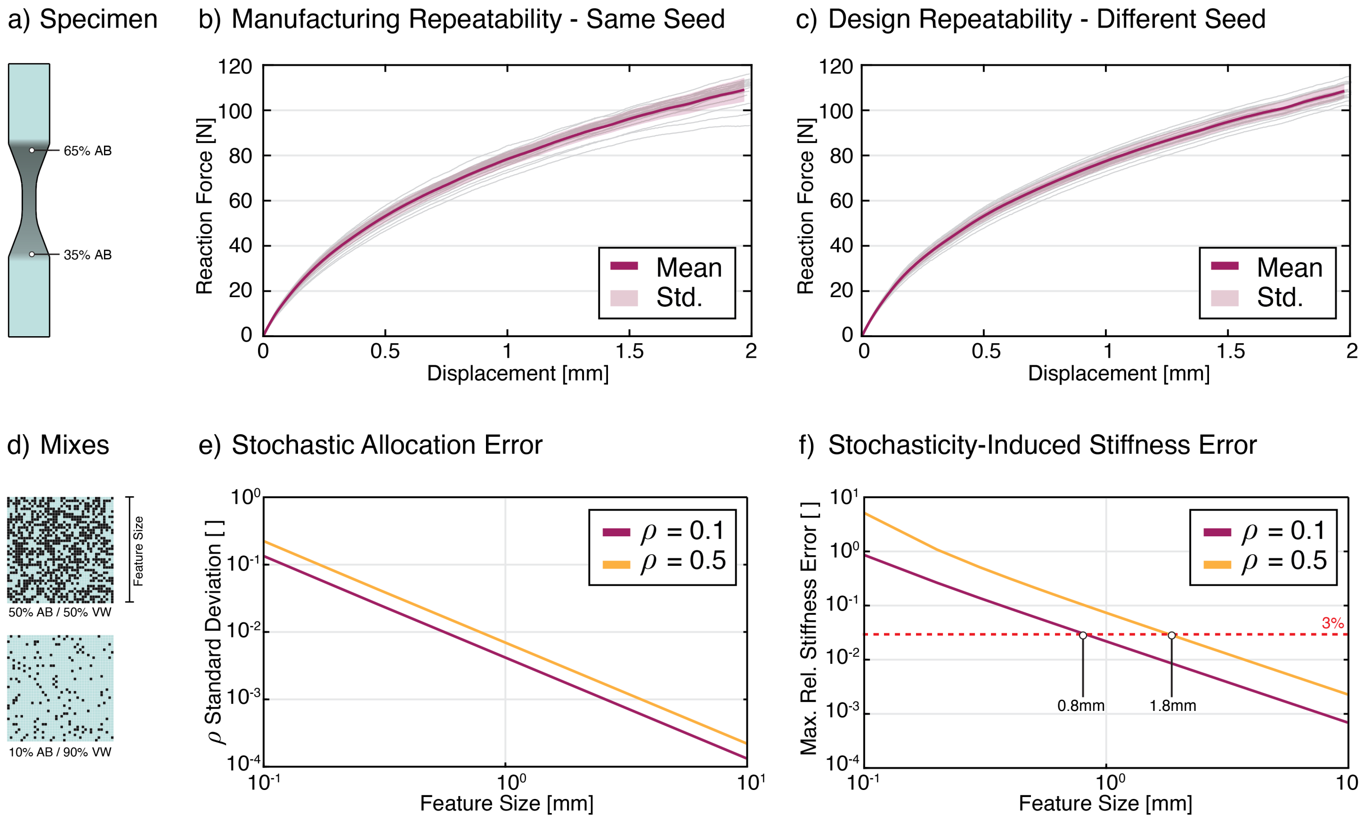

Supplementary Figure 22 (a) Graded specimen for repeatability tests. (b) Test data for 20 samples generated with the same voxel layout to quantify manufacturing accuracy. (c) Test data for 20 samples generated with different voxel layouts to quantify variability due to stochastic base material assignment. (d) Random stochastic digital materials for analytical derivation of expected mismatch due to stochasticity of the base material assignment. (e) Expected standard deviation in the mix ratio for different feature sizes. (f) Maximum stiffness mismatch induced by errors in the base material mix ratio for different feature sizes. Source data are provided as a Source Data file.

B.4 Neural Network Prediction Accuracy

The prediction accuracy of ANN_E is shown in Supplementary Figure 23. For twelve base material mix ratios on the full spectrum between full AB and full VW, the estimated stiffness is compared to the target stiffness linearly interpolated from the dataset. It is shown over all temperature and time spans. For each base material mix ratio, the biggest error relative to the target in both positive and negative direction is indicated with a marker. It becomes visible that the points of higher errors show a tendency towards higher time spans. This is beneficial for the Prony Series fitting procedure, since an error at higher frequencies yields a lower relative error in the master curve and yields a more accurate material model. Further, the error magnitude is in the range of a few MPa, which is negligible compared to other error sources, for example manufacturing variability.

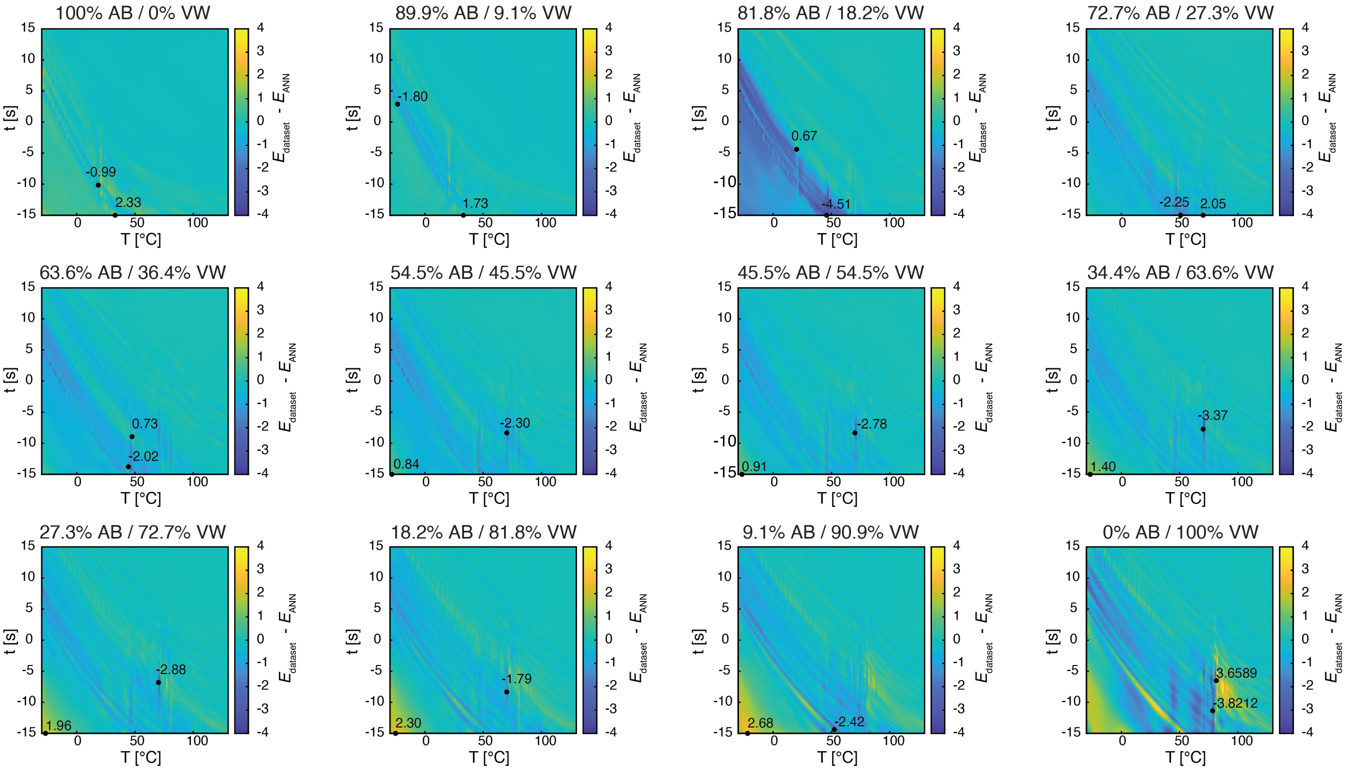

Supplementary Figure 23 Prediction accuracy of ANN_E. The difference between stiffness estimation and the underlying dataset is shown for twelve base material mix ratios for all temperatures and time spans. Source data are provided as a Source Data file.

The prediction accuracy of ANN_{mix} is shown in Supplementary Figure 24. For twelve base material mix ratios between the percolation threshold of 16.3% VW and full VW, the estimated mix ratio is compared to the target mix ratio. The error is shown as an absolute error over all temperatures and time spans. For each base material mix ratio, the maximum error is indicated with a marker. It is seen that the error over all mix ratios is below 1%. However, for all mix ratios, the accuracy decreases in ripple-like pattern at high temperatures and frequencies below 1 Hz. Since this pattern occurs in the region, where both constituent polymers are in the rubbery phase, a mismatch in base material mix ratio only has a minor effect on the material properties. Even for the highest error of 5.7% at a mix ratio at the percolation threshold, the mismatch in estimated stiffness when applying this error is below 0.5MPa. The ANN_{mix} is thus considered to provide a well-posed estimator for the base material mix ratio given target requirements.

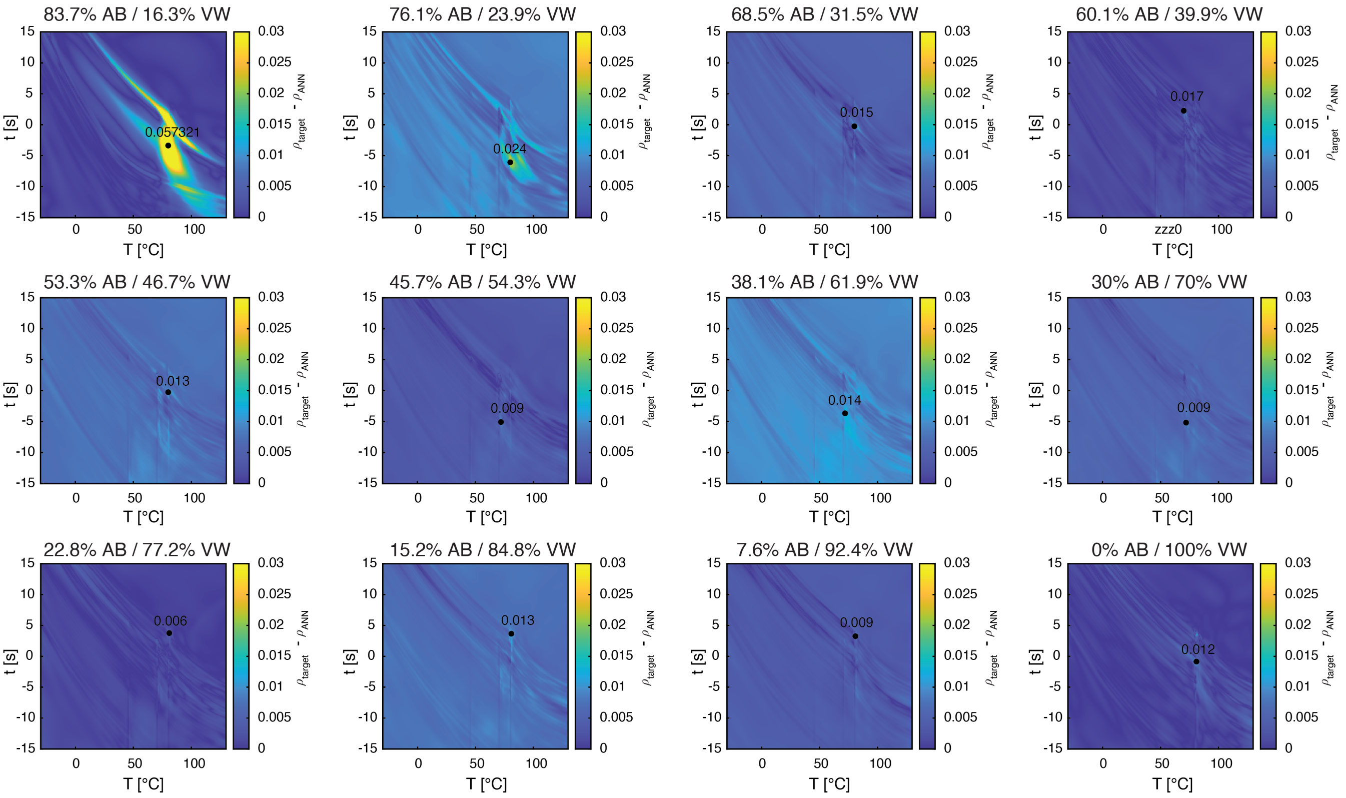

Supplementary Figure 24 Prediction accuracy of ANN_{mix}. The difference between the estimated base material mix ratio and the target base material mix ratio is shown for twelve base material mix ratios between the percolation threshold and full VW for all temperatures and time spans. Source data are provided as a Source Data file.

B.5 Orthosis Fatigue Testing

The orthosis shell material is tested under cyclic loading using the Instron ElectroPuls E3000 (Instron, Norwood, MA, USA) tensile testing machine with an Instron Dynacell 2527 having a range of ±5kN. The sample is a type IV dogbone sample according to ASTM D638 with rigid ends and graded interfaces according to Section A.1.1.1. The sample is tested over 10,000 cycles with a sinusoidal loading under ambient conditions. The mean strain is 1.5% and the amplitude is 0.5% relative to the dogbones strain gauge of 33mm. The results in Supplementary Figure 25 show no material deterioration and even a slight stiffness increase over time. This could be due to changes in the ambient conditions. The results show that the polymeric material is suitable for use in cyclic applications, such as a wrist orthosis.

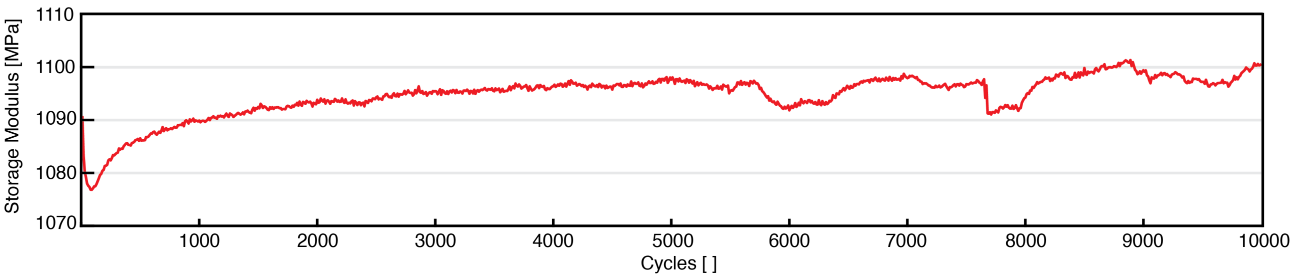

Supplementary Figure 25 Storage modulus of a dogbone made of orthosis shell material over 10,000 cycles. Source data are provided as a Source Data file.

C. Supplementary Figures

C.1 Prony Series Fitting

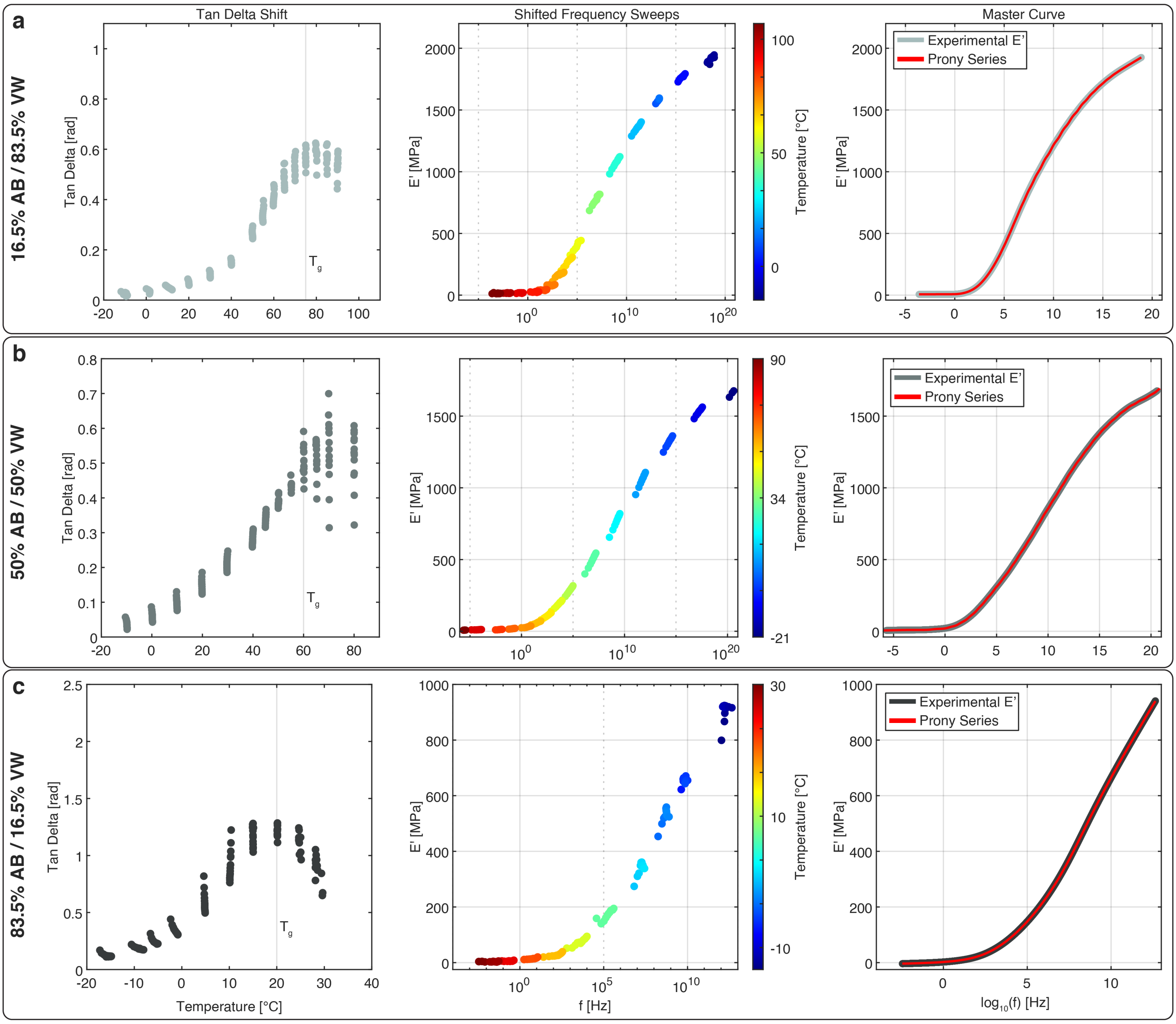

Supplementary Figure 26 Experimentally determined material properties consisting of $tan(\delta)$ in the first column, the shifted storage moduli from the frequency sweeps in the second column and the finalized experimental storage moduli and their respective Prony series in the third column. Source data are provided as a Source Data file.

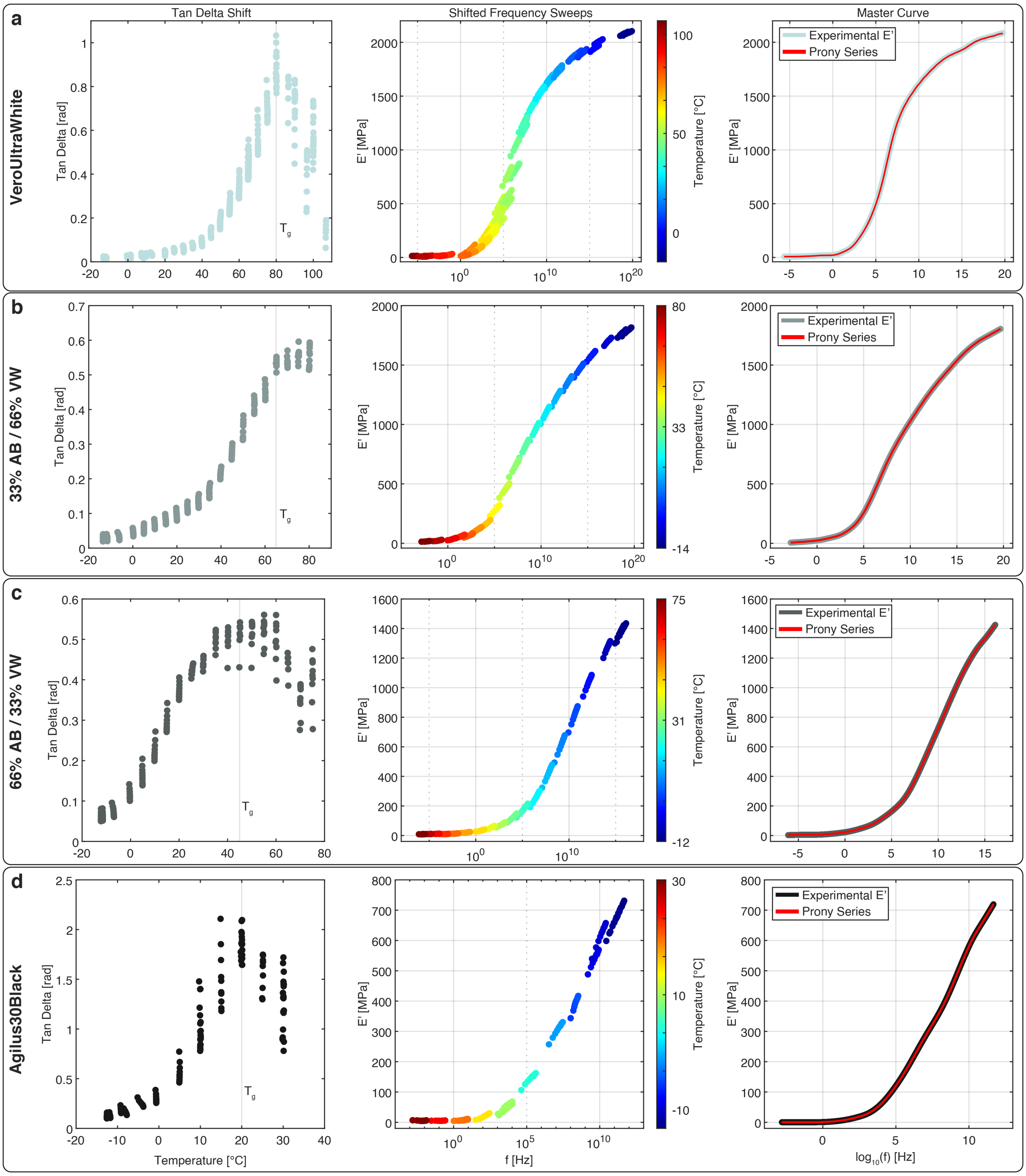

Supplementary Figure 27 Experimentally determined material properties consisting of $\tan(\delta)$ in the first column, the shifted storage moduli from the frequency sweeps in the second column and the finalized experimental storage moduli and their respective Prony series in the third column. Source data are provided as a Source Data file.

C.2 Damper Simulation Results

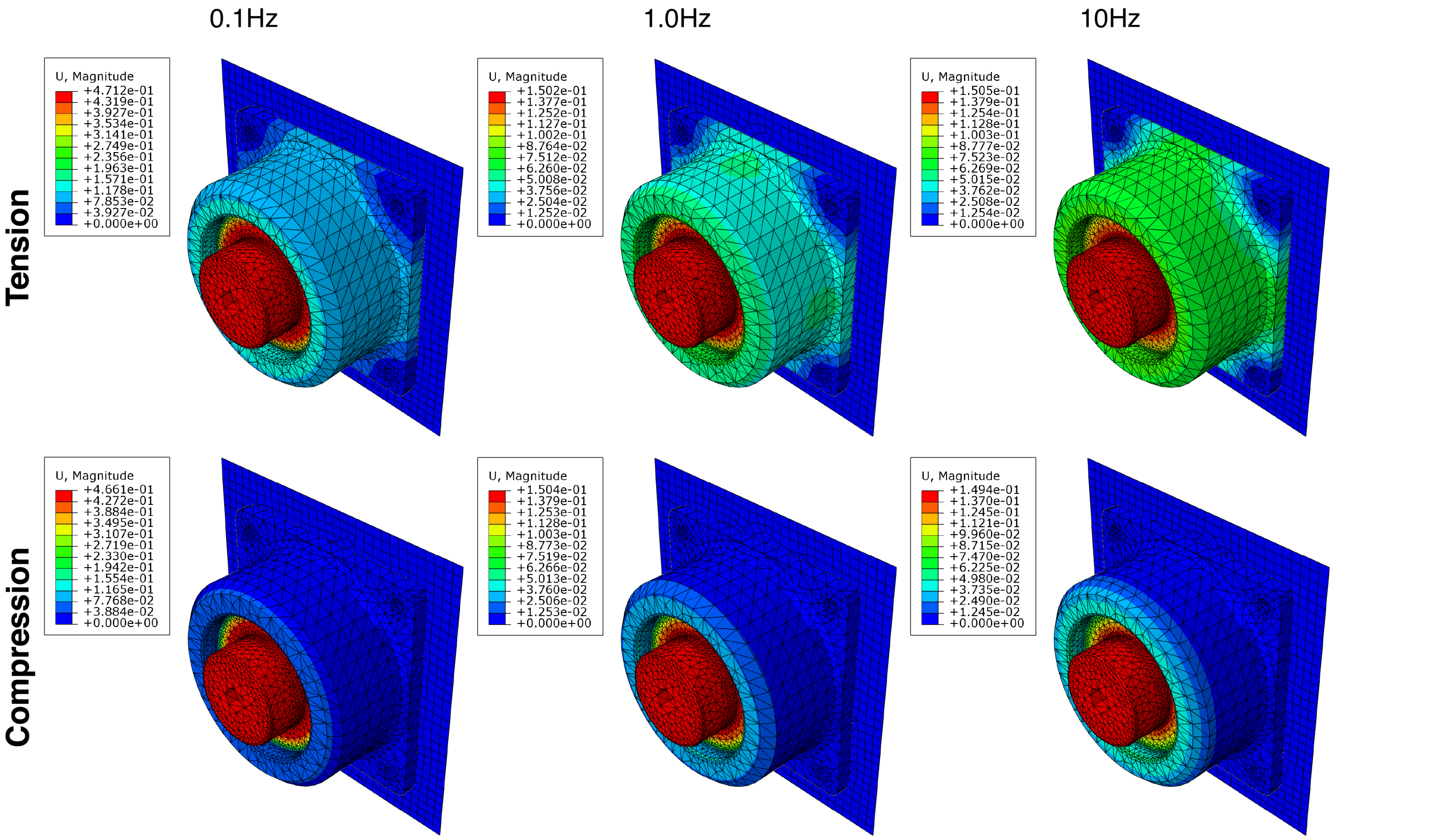

Supplementary Figure 28 Displacement magnitude(smoothed) of the second cycle of the hysteresis simulation for all three frequencies at 15℃.

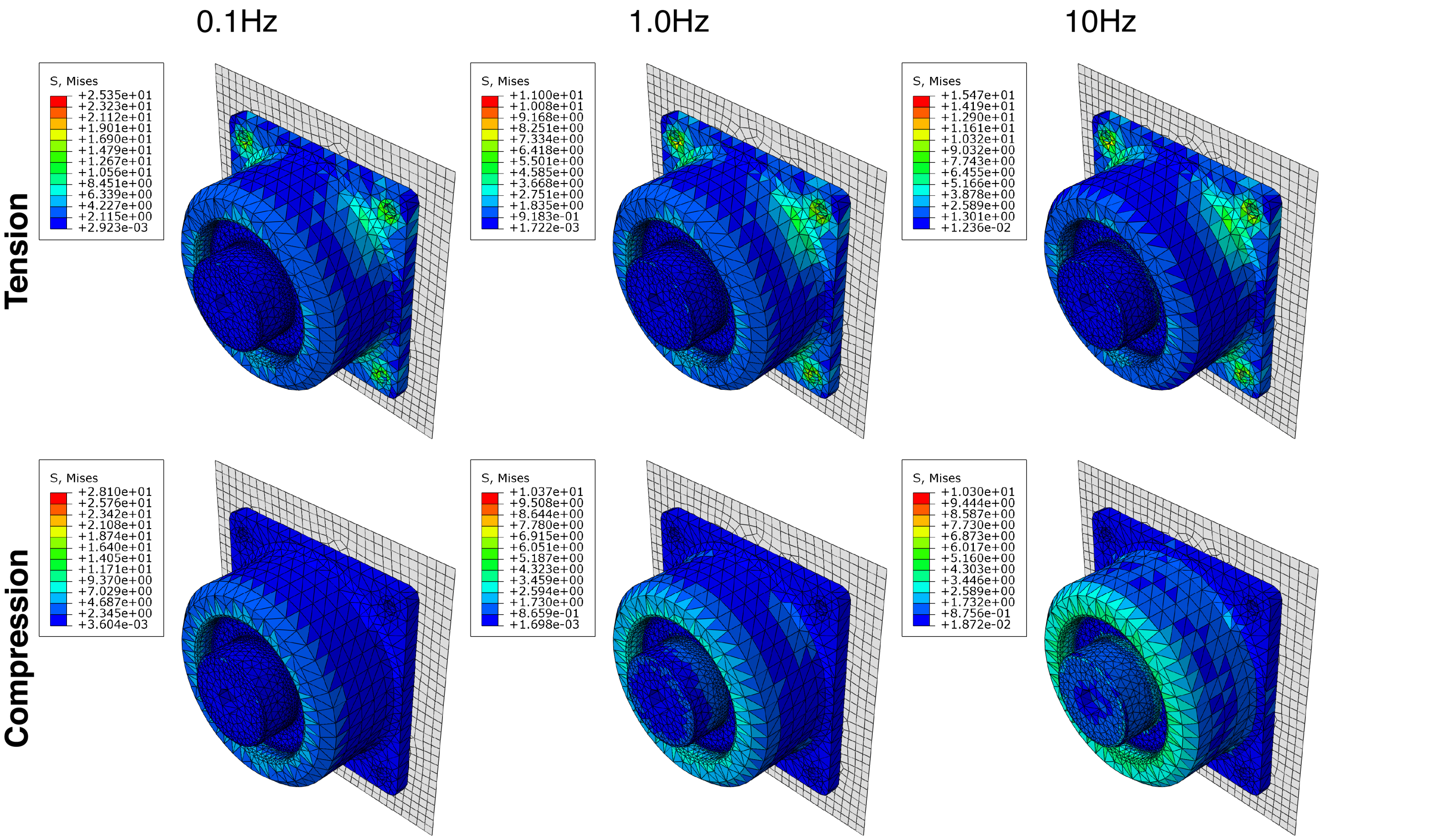

Supplementary Figure 29 Resulting von Mises stress (not averaged) of the second cycle of the hysteresis simulation for all three frequencies at 15℃.

C.3 Orthosis Simulation Results

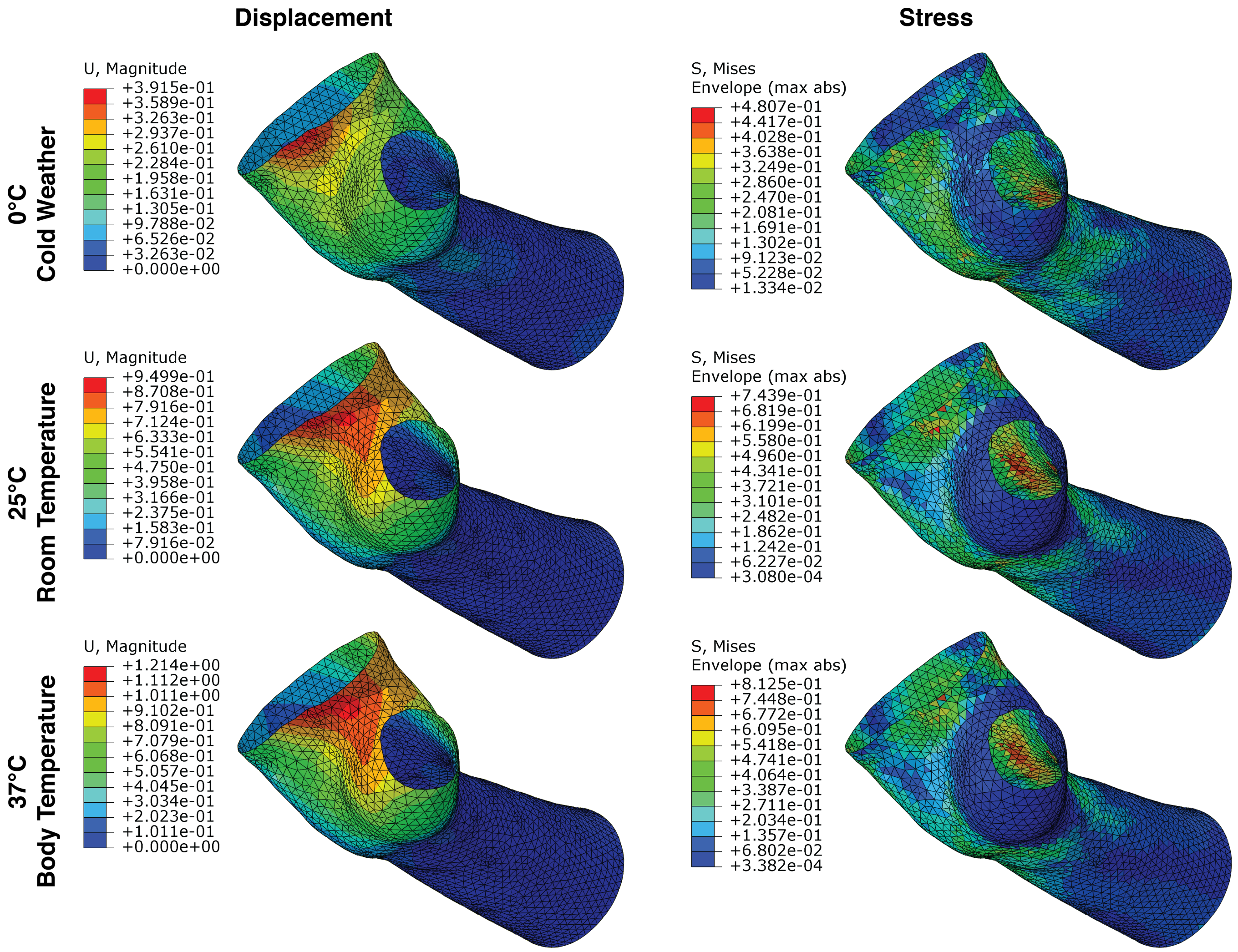

Supplementary Figure 30 Displacement magnitude (smoothed) and resulting von Mises stress after wrist flexion. The simulation is performed by a force application of -15N to the hand control point in angular direction at three different temperatures.

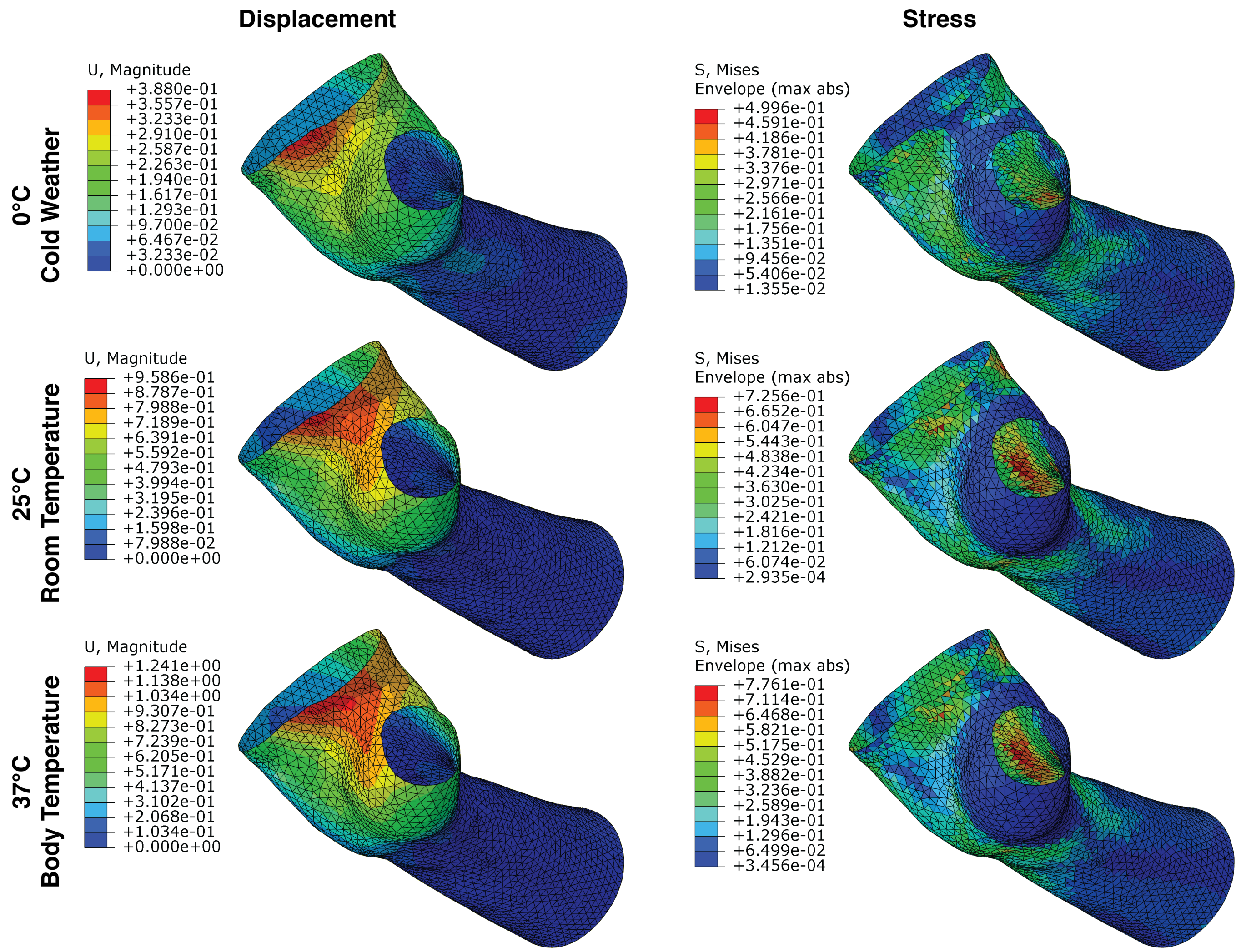

Supplementary Figure 31 Displacement magnitude (smoothed) and resulting von Mises stress after wrist extension. The simulation is performed by a force application of 15N to the hand control point in angular direction at three different temperatures.

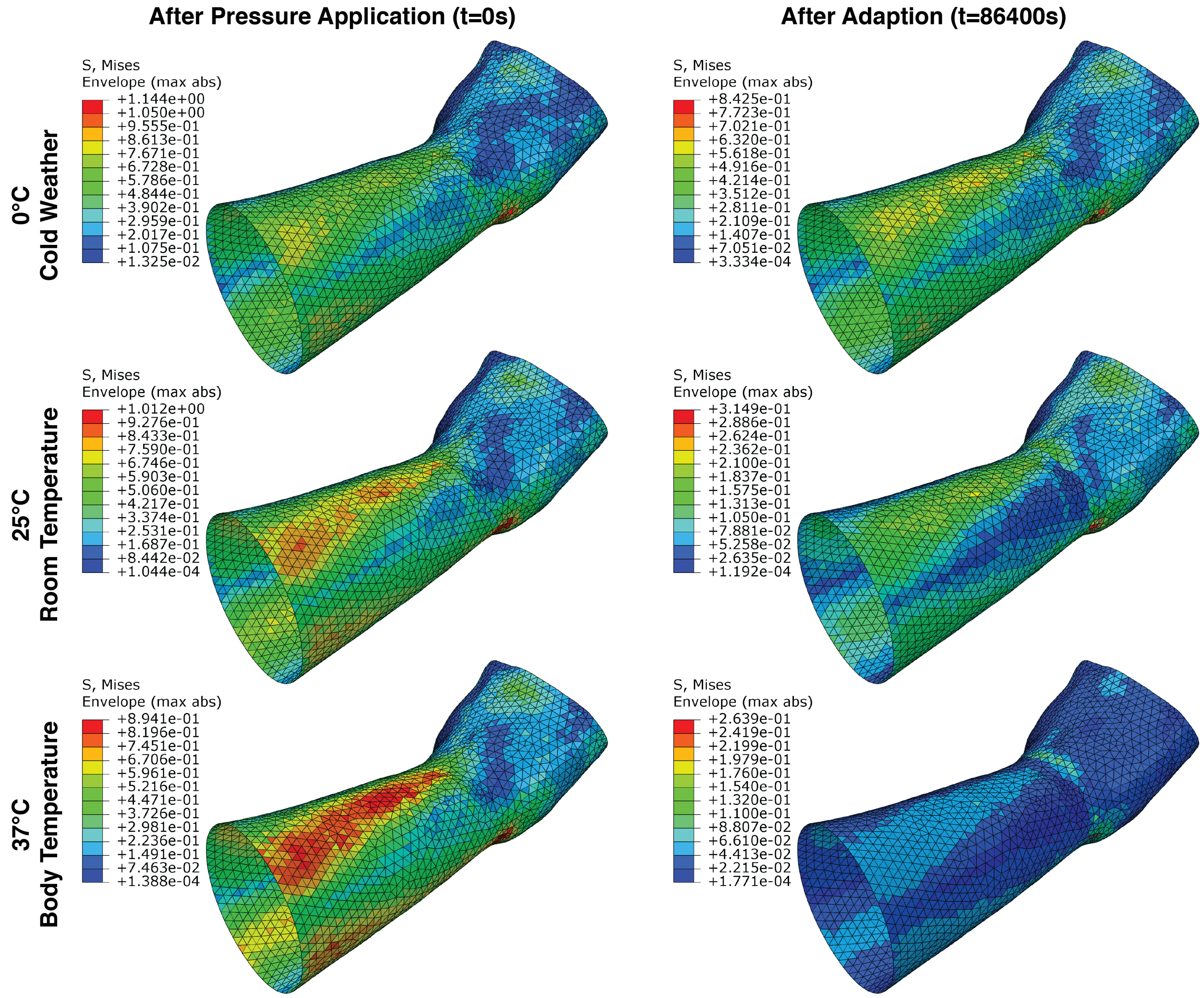

Supplementary Figure 32 Resulting stress shown after application of 0.02MPa inside pressure to the forearm and after relaxation of one day. A significant stress reduction of about 70% can be observed at 25℃ and 37℃.

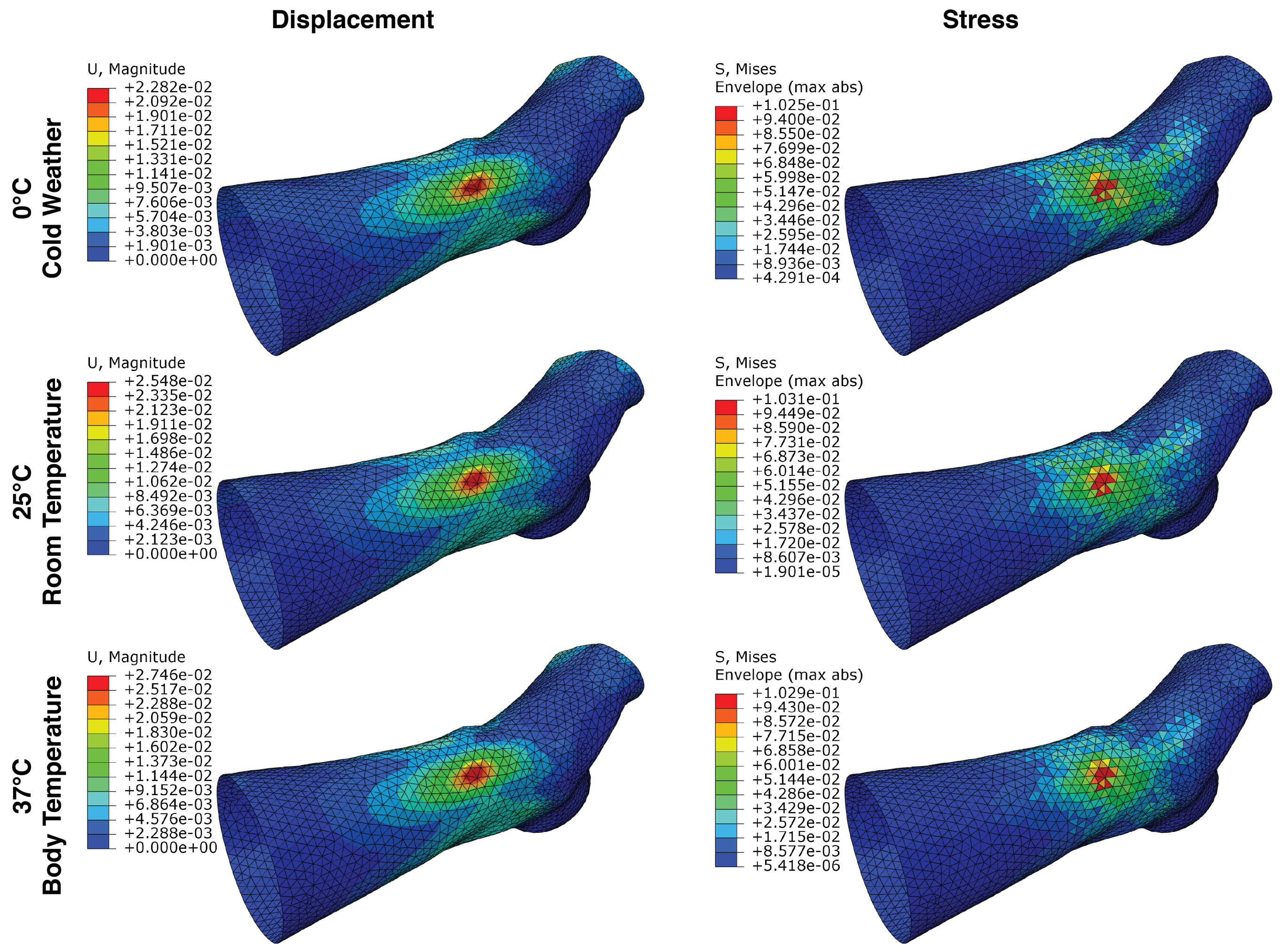

Supplementary Figure 33 Displacement magnitude (smoothed) and resulting stress of the impact simulation. Impact is simulated by applying a normal force to the shell with a magnitude of 120N over a time of 0.01s. Both the displacement and stress show a very low value indicating a good impact resistance of the orthosis.

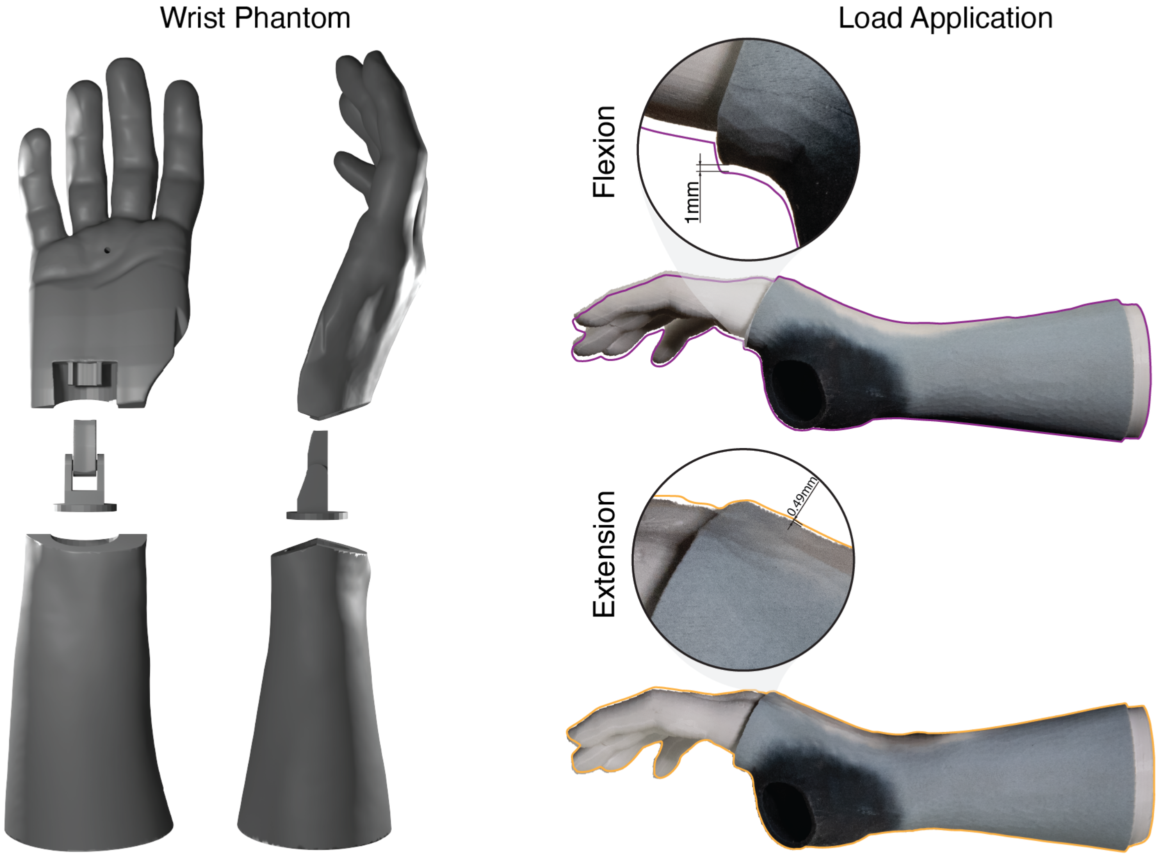

Supplementary Figure 34 Left: Rendered explosion view of wrist phantom used for testing flexion and extension. Right: Deformation of the wrist orthosis after a load application of 1.5kg to the wrist phantom. The deformation is shown in the close-up images. The phantom geometry is derived from a 3D scan provided by Theebadge/printables.com.

D. Supplementary Tables

D.1 Fitted Material Models

Supplementary Table 6 Hyperelastic data for the four materials used to conduct the percolation-based fitting procedure.

	VW	16.5% VW	33% VW	50% VW	66% VW	83.5% VW	AB
ε [mm/mm]	σ_{Hyper} [MPa]	σ_{Hyper} [MPa]	σ_{Hyper} [MPa]	σ_{Hyper} [MPa]	σ_{Hyper} [MPa]	σ_{Hyper} [MPa]	σ_{Hyper} [MPa]
0.004	0.0339	0.0245	0.0225	0.0229	0.0122	0.0049	0.0012
0.008	0.0678	0.0461	0.0449	0.0420	0.0243	0.0058	0.0024
0.012	0.1016	0.0711	0.0674	0.0610	0.0365	0.0121	0.0036
0.016	0.1355	0.0952	0.0899	0.0806	0.0486	0.0147	0.0048
0.020	0.1694	0.1153	0.1124	0.0995	0.0608	0.0178	0.0060
0.023	0.1953	0.1386	0.1304	0.1187	0.0711	0.0220	0.0068
0.027	0.2230	0.1642	0.1484	0.1371	0.0802	0.0251	0.0079
0.030	0.2516	0.1861	0.1674	0.1565	0.0886	0.0295	0.0098
0.033	0.2783	0.2085	0.1850	0.1751	0.0966	0.0332	0.0113
0.037	0.3054	0.2333	0.2037	0.1941	0.1060	0.0361	0.0119
0.040	0.3334	0.2570	0.2207	0.2116	0.1144	0.0407	0.0126
0.043	0.3614	0.2798	0.2394	0.2295	0.1245	0.0445	0.0141
0.047	0.3883	0.3013	0.2565	0.2498	0.1322	0.0489	0.0158
0.050	0.4154	0.3256	0.2741	0.2668	0.1409	0.0516	0.0168
0.053	0.4435	0.3491	0.2915	0.2864	0.1502	0.0540	0.0183
0.057	0.4702	0.3722	0.3097	0.3039	0.1603	0.0624	0.0195
0.060	0.4959	0.3963	0.3273	0.3229	0.1684	0.0619	0.0199
0.063	0.5231	0.4188	0.3448	0.3406	0.1770	0.0662	0.0208
0.067	0.5503	0.4416	0.3645	0.3583	0.1855	0.0712	0.0218
0.070	0.5773	0.4652	0.3791	0.3768	0.1934	0.0753	0.0232
0.073	0.6045	0.4880	0.3971	0.3959	0.2025	0.0766	0.0243
0.077	0.6301	0.5110	0.4155	0.4126	0.2130	0.0822	0.0258
0.080	0.6577	0.5357	0.4322	0.4314	0.2199	0.0860	0.0263
0.083	0.6850	0.5563	0.4491	0.4482	0.2274	0.0882	0.0274
0.087	0.7101	0.5814	0.4682	0.4673	0.2378	0.0937	0.0288
0.090	0.7360	0.6038	0.4847	0.4863	0.2459	0.0962	0.0294
0.093	0.7616	0.6248	0.5013	0.5032	0.2545	0.0991	0.0314
0.097	0.7894	-	0.5191	0.5204	0.2642	0.1023	0.0309
0.100	0.8155	-	0.5372	0.5391	0.2728	0.1069	0.0338

Supplementary Table 7 Relevant temperature shifting data and Prony element data for the experimental samples.

**VW**		**33% VW**		**66% VW**		**AB**	
$k_0\ [N/mm]$	8.469	$k_0\ [N/mm]$	5.618	$k_0\ [N/mm]$	3.039	$k_0\ [N/mm]$	0.3
T_g [°C]	80	T_g [°C]	70	T_g [°C]	45	T_g [°C]	20
$\nu\ [-]$	0.5	$\nu\ [-]$	0.5	$\nu\ [-]$	0.5	$\nu\ [-]$	0.5
$C_1\ [-]$	17.44	$C_1\ [-]$	17.44	$C_1\ [-]$	17.44	$C_1\ [-]$	17.44
$C_2\ [-]$	51.6	$C_2\ [-]$	51.6	$C_2\ [-]$	51.6	$C_2\ [-]$	51.6
$C_3\ [-]$	18617	$C_3\ [-]$	20075	$C_3\ [-]$	22128	$C_3\ [-]$	25011
$k_i\ [-]$	$\tau_i\ [s]$	$k_i\ [-]$	$\tau_i\ [s]$	$k_i\ [-]$	$\tau_i\ [s]$	$k_i\ [-]$	$\tau_i\ [s]$
1.307E-02	6.530E-20	2.031E-02	2.710E-20	5.209E-02	1.060E-16	6.699E-02	1.980E-12
9.597E-03	7.380E-19	1.767E-02	1.560E-19	4.020E-02	6.170E-16	2.043E-02	4.500E-12
1.173E-02	6.230E-18	1.698E-02	8.780E-19	4.350E-02	3.850E-15	4.223E-02	8.740E-12
1.695E-02	3.810E-17	2.193E-02	4.850E-18	5.182E-02	2.000E-14	4.201E-02	2.390E-11
1.797E-02	1.750E-16	2.506E-02	2.490E-17	5.803E-02	1.030E-13	5.065E-02	6.570E-11
1.322E-02	9.040E-16	2.927E-02	1.280E-16	6.471E-02	5.150E-13	5.403E-02	1.510E-10
1.276E-02	5.420E-15	3.300E-02	6.360E-16	6.745E-02	2.470E-12	5.331E-02	3.370E-10
1.610E-02	3.330E-14	3.504E-02	3.250E-15	6.863E-02	1.170E-11	5.342E-02	7.250E-10
2.291E-02	1.850E-13	3.548E-02	1.700E-14	6.643E-02	5.380E-11	5.403E-02	1.610E-09
2.798E-02	1.050E-12	3.909E-02	9.300E-14	6.376E-02	2.470E-10	5.226E-02	3.690E-09
3.118E-02	6.270E-12	4.144E-02	5.030E-13	6.296E-02	1.060E-09	4.712E-02	9.110E-09
3.719E-02	4.000E-11	4.557E-02	2.710E-12	5.881E-02	4.410E-09	4.289E-02	2.420E-08
4.277E-02	2.570E-10	4.737E-02	1.470E-11	5.490E-02	1.760E-08	4.372E-02	6.380E-08
4.712E-02	1.570E-09	4.801E-02	7.730E-11	4.684E-02	6.790E-08	4.584E-02	1.640E-07
5.780E-02	8.430E-09	4.961E-02	4.040E-10	3.663E-02	2.570E-07	4.578E-02	4.110E-07
7.327E-02	3.830E-08	5.177E-02	2.040E-09	2.543E-02	9.500E-07	4.109E-02	9.730E-07
9.614E-02	1.500E-07	5.646E-02	9.620E-09	1.717E-02	3.230E-06	3.873E-02	2.170E-06
9.929E-02	5.660E-07	6.075E-02	4.280E-08	1.235E-02	7.750E-06	3.513E-02	5.000E-06
7.822E-02	1.970E-06	6.280E-02	1.800E-07	1.291E-02	1.770E-05	2.844E-02	1.100E-05
6.460E-02	7.300E-06	5.860E-02	7.390E-07	1.216E-02	4.230E-05	2.717E-02	2.210E-05
5.135E-02	2.790E-05	5.252E-02	2.950E-06	1.627E-02	1.260E-04	2.590E-02	4.840E-05
4.078E-02	1.030E-04	4.245E-02	1.220E-05	1.298E-02	4.420E-04	3.056E-03	9.980E-05
3.335E-02	3.970E-04	3.090E-02	5.110E-05	1.140E-02	1.590E-03	1.632E-02	9.980E-05
2.903E-02	1.480E-03	2.202E-02	2.350E-04	8.599E-03	7.260E-03	3.301E-04	1.010E-04
1.902E-02	5.210E-03	1.700E-02	1.120E-03	7.503E-03	2.960E-02	2.131E-02	2.290E-04
1.062E-02	2.310E-02	9.866E-03	5.680E-03	6.571E-03	1.190E-01	1.666E-02	6.850E-04
1.082E-02	9.220E-02	8.042E-03	3.910E-02	5.132E-03	5.160E-01	9.638E-03	2.450E-03
5.662E-03	3.750E-01	7.011E-03	2.350E-01	4.082E-03	2.700E+00	9.343E-03	9.140E-03
2.388E-03	5.610E+01	4.224E-03	1.690E+00	4.019E-03	1.850E+01	6.651E-03	4.000E-02
1.397E-03	3.330E+02	3.458E-03	1.560E+01	2.893E-03	1.990E+02	3.872E-03	1.990E-01
1.683E-03	3.670E+03	3.228E-03	1.820E+02	1.660E-03	2.110E+05	1.257E-03	7.830E-01

Supplementary Table 8 Relevant temperature shifting data and Prony element data for the experimental samples.

**16.5% VW**		**50% VW**		**83.5% VW**	
$k_0\ [N/mm]$	1.0759	$k_0\ [N/mm]$	5.337	$k_0\ [N/mm]$	6.723
$T_g\ [°C]$	20	$T_g\ [°C]$	60	$T_g\ [°C]$	75
$\nu\ [-]$	0.5	$\nu\ [-]$	0.5	$\nu\ [-]$	0.5
$C_1\ [-]$	17.44	$C_1\ [-]$	17.44	$C_1\ [-]$	17.44
$C_2\ [-]$	51.6	$C_2\ [-]$	51.6	$C_2\ [-]$	51.6
$C_3\ [-]$	24223	$C_3\ [-]$	20976	$C_3\ [-]$	19294
$k_i\ [-]$	$\tau_i\ [s]$	$k_i\ [-]$	$\tau_i\ [s]$	$k_i\ [-]$	$\tau_i\ [s]$
7.090E-02	2.540E-13	3.101E-02	1.600E-21	1.808E-02	1.820E-19
5.330E-05	4.070E-13	2.262E-02	1.450E-20	1.480E-02	7.790E-19
6.487E-02	1.080E-12	2.179E-02	1.520E-19	1.371E-02	2.950E-18
5.225E-02	3.960E-12	2.706E-02	1.480E-18	1.049E-02	8.850E-18
5.121E-02	1.180E-11	3.283E-02	1.150E-17	1.573E-02	2.670E-17
5.121E-02	3.510E-11	3.523E-02	9.110E-17	2.264E-02	1.260E-16
5.608E-02	1.060E-10	3.973E-02	6.810E-16	2.700E-02	7.280E-16
5.763E-02	3.380E-10	4.538E-02	4.700E-15	3.142E-02	4.330E-15
5.550E-02	1.010E-09	4.590E-02	3.150E-14	3.964E-02	2.930E-14
5.025E-02	2.770E-09	4.886E-02	2.080E-13	4.859E-02	2.390E-13
4.992E-02	6.560E-09	5.412E-02	1.320E-12	5.407E-02	2.090E-12
6.247E-02	1.910E-08	5.318E-02	7.970E-12	5.944E-02	1.840E-11
4.887E-02	5.850E-08	5.259E-02	4.980E-11	6.395E-02	1.600E-10
4.440E-02	1.520E-07	5.565E-02	3.090E-10	6.590E-02	1.330E-09
4.741E-02	4.640E-07	5.436E-02	1.800E-09	6.661E-02	9.740E-09
3.916E-02	1.470E-06	5.101E-02	1.100E-08	6.625E-02	6.050E-08
3.429E-02	4.330E-06	5.086E-02	6.820E-08	6.536E-02	3.160E-07
2.904E-02	1.230E-05	4.769E-02	4.100E-07	6.012E-02	1.410E-06
2.726E-02	3.430E-05	4.322E-02	2.610E-06	5.358E-02	5.480E-06
2.158E-02	9.660E-05	4.230E-02	1.680E-05	5.009E-02	2.150E-05
1.596E-02	2.210E-04	4.034E-02	1.075E-04	3.797E-02	7.990E-05
1.869E-02	5.898E-04	3.614E-02	7.086E-04	3.167E-02	2.561E-04
1.374E-02	1.804E-03	2.838E-02	4.996E-03	2.361E-02	7.836E-04
1.111E-02	5.111E-03	1.970E-02	4.022E-02	2.081E-02	2.280E-03
8.763E-03	1.835E-02	1.013E-02	4.367E-01	1.353E-02	7.026E-03
4.566E-03	6.695E-02	4.461E-03	1.639E+01	8.400E-03	1.852E-02
3.932E-03	1.921E-01	6.450E-04	1.313E+04	6.046E-03	4.448E-02
3.657E-03	1.074E+00	9.362E-04	1.051E+05	4.796E-03	1.339E-01
1.229E-03	5.235E+00	7.895E-04	7.452E+05	7.450E-07	1.580E-01
1.675E-03	1.990E+01	5.990E-07	2.514E+11	1.765E-03	3.685E-01
1.242E-03	1.103E+02	2.300E-06	1.317E+12	4.636E-04	1.049E+02

E. Supplementary References

1. Mueller, J., Shea, K. & Daraio, C. Mechanical properties of parts fabricated with inkjet 3D printing through efficient experimental design. *Mater Des* **86**, 902–912 (2015).
2. Ferry, J. D. *Viscoelastic Properties of Polymers*. (John Wiley & Sons, 1980).
3. Wirth, M., Chapuis, J. N. & Shea, K. Inverse Design of Stochastic, Voxelated Thermo-Viscoelastic Digital Materials. *STAMP* (2025) doi:https://doi.org/10.5281/zenodo.17296598.
4. Adam H. Aitkenhead. Mesh Voxelisation. https://www.mathworks.com/matlabcentral/fileexchange/27390-mesh-voxelisation (2024).
5. Patil, S. & Ravi, B. Voxel-based Representation, Display and Thickness Analysis of Intricate Shapes. in *Ninth International Conference on Computer Aided Design and Computer Graphics (CAD-CG'05)* 415–422 (IEEE). doi:10.1109/CAD-CG.2005.86.
6. Kingma, D. P. & Ba, J. Adam: A Method for Stochastic Optimization. http://arxiv.org/abs/1412.6980 (2017).
7. Howell, D. M., Bechmann, S. & Underwood, P. J. *Wrist Splint*. (2024).
8. Zhou, M. *et al.* The design and manufacturing of a Patient-Specific wrist splint for rehabilitation of rheumatoid arthritis. *Mater Des* **238**, 112704 (2024).
9. Kim, H. & Jeong, S. Case study: Hybrid model for the customized wrist orthosis using 3D printing. *Journal of Mechanical Science and Technology* **29**, 5151–5156 (2015).
10. Cazon, A., Kelly, S., Paterson, A. M., Bibb, R. J. & Campbell, R. I. Analysis and comparison of wrist splint designs using the finite element method: Multi-material three-dimensional printing compared to typical existing practice with thermoplastics. *Proc Inst Mech Eng H* **231**, 881–897 (2017).
11. Vanswearingen, J. M. Measuring Wrist Muscle Strength. *Journal of Orthopaedic & Sports Physical Therapy* **4**, 217–228 (1983).
12. Delp, S. L., Grierson, A. E. & Buchanan, T. S. Maximum isometric moments generated by the wrist muscles in flexion-extension and radial-ulnar deviation. *J Biomech* **29**, 1371–1375 (1996).
13. Kao, H.-K. *et al.* Application of Simulated Arms with Real-Time Pressure Monitor in Casting and Splinting by Physiological Sensors. *Sensors* **21**, 5681 (2021).
14. Zaidi, S. R. H. & Sharma, S. *Pressure Ulcer*. (2024).
15. Grey, J. E., Harding, K. G. & Enoch, S. Pressure ulcers. *BMJ* **332**, 472–475 (2006).
16. Plagenhoef, S., Evans, F. G. & Abdelnour, T. Anatomical Data for Analyzing Human Motion. *Res Q Exerc Sport* **54**, 169–178 (1983).
17. Lewek, M. D., Poole, R., Johnson, J., Halawa, O. & Huang, X. Arm swing magnitude and asymmetry during gait in the early stages of Parkinson's disease. *Gait Posture* **31**, 256–60 (2010).
18. Heinemann, P. & Kasperski, M. Damping Induced by Walking and Running. *Procedia Eng* **199**, 2826–2831 (2017).
19. Prusa Research. Hand and forearm natural position (Scan). *Printables* https://www.printables.com/model/349380-hand-and-forearm-natural-position-scan (2023).
20. Stratasys Ltd. *Datasheet Agilus30*. https://www.stratasys.com/globalassets/materials/materials-catalog/polyjet-materials/agilus30/mds_pj_agilus30_0121b.pdf (2021).
21. Stratasys Ltd. *Datasheet VeroUltra*. https://www.stratasys.com/siteassets/materials/materials-catalog/polyjet-materials/veroultra/mds_pj_veroultra-for-j55_0321b-1.pdf?v=48e03c (2021).
22. De Gennes, P. G. On a relation between percolation theory and the elasticity of gels. *Journal de Physique Lettres* **37**, 1–2 (1976).

23. Soussou, J. E., Moavenzadeh, F. & Gradowczyk, M. H. Application of Prony Series to Linear Viscoelasticity. *Transactions of the Society of Rheology* **14**, 573–584 (1970).

24. Ho, C., Bao, J. & Xu, J. Thermomechanical Properties of Polyjet Voxel-Printed Parts and the Effect of Percolation. *3D Print Addit Manuf* **11**, 1799–1809 (2024).

25. Mueller, J., Courty, D., Spielhofer, M., Spolenak, R. & Shea, K. Mechanical Properties of Interfaces in Inkjet 3D Printed Single- and Multi-Material Parts. *3D Print Addit Manuf* **4**, 193–199 (2017).

26. Meisel, N. A., Dillard, D. A. & Williams, C. B. Impact of material concentration and distribution on composite parts manufactured via multi-material jetting. *Rapid Prototyp J* **24**, 872–879 (2018).
